# Gut virome remodeling after fecal microbiota transplantation is associated with virus–bacteria co-variation and metabolic response in obesity and type 2 diabetes

**DOI:** 10.1080/19490976.2026.2746820

**Published:** 2026-10-23

**Authors:** Hui Jiang, Ye Peng, Brian Lam, Keli Yang, Yu Lin, Joyce W. Y. Mak, Yee Kit Tse, Francis K. L. Chan, Zhilu Xu, Siew C. Ng

**Affiliations:** a Microbiota I-Center (MagIC), Hong Kong SAR, China; b Department of Psychiatry, The Chinese University of Hong Kong, Hong Kong SAR, China; c Jockey Club School of Public Health and Primary Care, Faculty of Medicine, The Chinese University of Hong Kong, Hong Kong SAR, China; d Department of Medicine and Therapeutics, The Chinese University of Hong Kong, Hong Kong SAR, China; e State Key Laboratory of Digestive Disease, Institute of Digestive Disease, LKS Institute of Health Science, The Chinese University of Hong Kong, Hong Kong SAR, China; f Center for Gut Microbiota Research, Faculty of Medicine, The Chinese University of Hong Kong, Hong Kong SAR, China

**Keywords:** Gut virome, fecal microbiota transplantation, obesity, type 2 diabetes mellitus, viral engraftment, metabolic response

## Abstract

Fecal microbiota transplantation (FMT) modifies gut bacterial composition and improves metabolic outcomes in subjects with obesity and type 2 diabetes (ObT2), but bacterial changes alone do not fully explain the heterogeneity in clinical response. We performed virus-like particle (VLP)-enriched metagenomic sequencing coupled with bulk-DNA metagenomic sequencing on longitudinal fecal samples from a randomized controlled FMT trial in ObT2. Using random forest classification, post-FMT virome changes showed accuracy comparable to bacteriome changes (84.8% vs 81.4%) in distinguishing LDL responders from non-responders. Donor-derived viral contig engraftment was detected in 64.7% (11/17) of recipients receiving FMT alone and 58.8% (10/17) receiving FMT with lifestyle intervention (LSI), versus 5.6% (1/18) in the sham with LSI group. FMT was associated with increased virome diversity, compositional shifts toward the donor virome, and coordinated virus–bacteria associations, with most responsive viral contigs classified as *Caudoviricetes*. FMT-responsive viral contigs that were shared between the two FMT groups were predicted to infect *Roseburia* and *Bifidobacterium*, while contigs associated with clinical responses were predicted to infect *Escherichia*, *Roseburia*, *Blautia*, and *Enterococcus*. These findings suggest that the gut virome is a complementary ecological component associated with FMT responses and metabolic outcomes in ObT2.

## Introduction

The gut microbiome is recognized as a key mediator of metabolic dysfunction in obesity and type 2 diabetes mellitus (T2DM),^1-3^ with microbiome-targeted therapies offering a promising therapeutic avenue.^4-7^ In our previous randomized controlled trial, repeated FMT promoted sustained engraftment of donor-derived microbiota and was associated with improvements in low-density lipoprotein (LDL) cholesterol and liver steatosis in obese individuals with T2DM.^8^

However, the gut microbiome extends beyond bacteria. The gut virome, composed predominantly of bacteriophages that infect bacteria, is increasingly recognized as an active ecological agent capable of reshaping microbial community structure through lytic and lysogenic cycles.^9-13^ Our group previously demonstrated that T2DM are associated with reduced gut virome diversity and disrupted virus–bacteria interactions.^14^ In the context of FMT, mounting evidence suggests that the gut virome is “not just a passenger” but may play active roles in treatment response.^15^ ^,^ ^16^ Bacteriophage composition has been linked to microbial engraftment and treatment response following FMT.^17^ ^,^ ^18^ A randomized controlled trial using sterile fecal filtrate transplantation showed that transfer of bacteriophages alone can transiently reshape gut microbiota and phage-microbe interactions.^19^ Moreover, preclinical studies have further demonstrated that fecal virome transplantation (FVT) alone can alleviate obesity and T2DM-related metabolic features in mouse models, supporting a functional role of the gut virome.^20^ ^,^ ^21^ More recently, human gut-derived virus-like particle (VLP) transplantation was shown to improve glucose tolerance while reshaping both the gut microbiota and virome in diet-induced obese mice, further strengthening the evidence that the gut virome may actively contribute to host metabolic regulation.^22^

Despite these advances, the contribution of the gut virome to FMT outcomes in human metabolic disease remains poorly defined. Existing human studies are limited in scale, and no randomized controlled trial has systematically characterized virome dynamics following FMT in obese T2DM populations. Building on our previous randomized controlled trial, we conducted VLP-enriched metagenomic sequencing on longitudinal fecal samples to characterize gut virome dynamics following repeated FMT. We hypothesized that: (1) repeated FMT is associated with sustained engraftment of donor-derived viral contigs; (2) FMT is associated with altered virus‒bacteria associations; and (3) specific viral contigs are associated with metabolic responses to FMT.

## Methods

### Study cohort

This study is a mechanistic extension of previously published RCT investigating the effects of FMT in obese subjects with type 2 diabetes (ObT2).^8^ The original trial recruited adults aged 18‒70 y with a body mass index (BMI) ≥28 kg/m^2^ and <45 kg/m^2^ and a diagnosis of T2DM for at least 3 months. Key exclusion criteria included recent use of antibiotics or probiotics, severe comorbidities, and contraindications to endoscopic procedures, as previously described. Participants were randomly assigned to receive FMT with lifestyle intervention (LSI), FMT alone, or sham with LSI. Per protocol, participants were asked to maintain stable doses of medications during follow-up, and any medication changes were recorded. FMT solution was prepared from six healthy lean donors. Clinical follow-up and biospecimen collection were performed at baseline and longitudinally up to week 24. Detailed study procedures, donor screening, FMT preparation, and clinical assessments have been described previously. All patients gave written informed consent. The original trial was approved by the Joint Chinese University of Hong Kong New Territories East Cluster Clinical Research Ethics Committee (2016.136-T). The study was registered with ClinicalTrials.gov (NCT03127696).

### Fecal VLP DNA extraction and sequencing

Archived stool samples collected during the original trial were used for VLP-enriched metagenomic sequencing. The detailed DNA extraction procedures can be found in our previous study.^14^ Briefly, stool (100–200 mg) was added to 400 μL saline-magnesium buffer and vortexed for 10 minutes. Suspensions were centrifuged at 2000 g to remove debris and cells. The supernatant was passed through 0.45- and 0.22-μm filters. To remove residual bacterial and host cell membranes, samples were treated with lysozyme (1 mg/mL at 37 °C for 30 minutes), followed by chloroform (0.2 × volume at room temperature for 10 minutes). Non-virus-protected DNA was removed by treatment with 1 U Baseline-Zero DNase (Epicenter), followed by heat inactivation at 65 °C for 10 minutes.

To extract nucleic acids from VLP, samples were cleaved with 4% sodium dodecyl sulfate plus 38 mg/mL proteinase K at 56 °C and then treated with CTAB buffer and phenol-chloroform-isoamyl alcohol (pH 8.0). The aqueous portion was washed once with an equal volume of chloroform, followed by concentration using a DNA Clean & Concentrator kit (Zymo Research). VLP DNA was amplified for 2 hours using Phi29 polymerase before sequencing. Sequencing libraries were prepared from 1 μg DNA per sample using the NEBNext Ultra DNA Library Prep Kit (Illumina) and sequenced on an Illumina NovaSeq 6000 platform with paired-end 150 bp reads, generating a median of 49.8 million reads per sample.

### Sequencing data processing, annotation, and quantification

Shotgun metagenomic reads were quality-filtered and decontaminated of human sequences using KneadData (https://bitbucket.org/biobakery/kneaddata/wiki/Home), which integrates Trimmomatic^23^ (v.0.39.1) and Bowtie2^24^ (v.2.3.4.3). Reads mapping to the human reference genome (hg38) were discarded. To assess the purity of VLP-enriched libraries, reads were taxonomically classified using Kraken2^25^ (v.2.1.3) against the PlusPF reference database (build 2026-06-26), with species-level abundances re-estimated using Bracken^26^ (v3.0.1). The proportions of bacterial, archaeal, fungal, viral, human, and unclassified reads were summarized to evaluate the extent of non-viral DNA contamination.

Paired-end VLP reads were assembled into contigs using MEGAHIT^27^ (v.1.0.3), yielding 349,698 contigs longer than 1000 bp. Contigs with an estimated completeness ≥50% and contamination ≤10% (medium quality or above; *n* = 3293) by CheckV^28^ (v1.0.1) were retained. The quality-controlled viral contigs were dereplicated at 99% average nucleotide identity (ANI) and 90% alignment fraction (AF) to generate a representative viral contigs set (*n* = 2588).

Representative viral contigs were taxonomically annotated using geNomad^29^ (v1.11.1; genomad_msa_v1.8) in end-to-end mode. Complementary protein-homology-based taxonomic annotations were obtained using DIAMOND^30^ (v2.1.10) searches against the RefSeq viral protein database (retrieved June 24, 2026), followed by taxonomic annotation using MEGAN^31^ (v6.25.10). Putative host associations were inferred using iPHoP (v1.3.3), and viral lifestyles were predicted using PhaTYP^32^ based on a database merged from “Sept_2021_pub_rw” and the MAGIC database (https://zenodo.org/records/13989345).^33^

To quantify the abundance of viral contigs, quality-filtered metagenomic reads from both bulk and VLP-enriched sequencing were competitively mapped against the representative viral contigs set using CoverM^34^ (v0.7.0) in genome mode with Strobealign (v0.17.0). Genome-level abundance was calculated as reads per kilobase million (RPKM). To avoid bias associated with Phi29 amplification of VLP libraries, RPKM values derived from bulk metagenomic reads were used and transformed into relative abundance for downstream analyzes.

Viral contigs detected in recipient post-FMT samples were classified into four categories based on their presence in the recipient baseline and the donor: (1) donor-derived: absent in recipient baseline but present in donor; (2) recipient-exclusive: present in recipient baseline but absent in donor; (3) co-existing: present in both recipient baseline and donor; and (4) unknown: absent in both recipient baseline and donor. The ≥20% threshold for donor-derived viral contigs was adapted from the microbiota engraftment framework of the original trial.^8^

### Machine learning classification of LDL response

To compare the classification performance of post-FMT changes in the gut virome versus the bacteriome for LDL response (≥0.5 mmol/L reduction at week 24), we performed random forest (RF) classification. RF was chosen for its ability to handle high-dimensional data, capture non-linear relationships, and provide interpretable variable importance measures. For both virome and bacteriome data, features present in at least 5% of samples with a mean relative abundance ≥0.01% were retained to remove low-abundance or rare taxa. Retained features were transformed using centered log-ratio (CLR), and *Δ* was calculated as the difference between week 24 and baseline (week 0). Recursive feature elimination (RFE) combined with RF was performed with 50 repeats to select stable features. Features selected in ≥50% of RFE repeats were defined as stable. Final RF classifiers were trained using these stable features with 500 trees per forest. Model performance was evaluated using stratified 5-fold cross-validation (CV), with the same folds applied to the virome and bacteriome models. Performance metrics included CV mean accuracy, Cohen's Kappa, and the area under the receiver operating characteristic curve (AUC), calculated from CV predictions to avoid overestimation. Sensitivity, specificity, and balanced accuracy were additionally calculated from cross-validated predictions to evaluate model performance under class imbalance. To assess whether differences in classification performance were influenced by feature-number asymmetry, an additional sensitivity analysis was performed by constructing both virome and bacteriome models using the same number of stable features selected by RFE. No class weighting or optimized classification threshold was used. Class imbalance was instead addressed using a resampling-based sensitivity analysis with the synthetic minority oversampling technique (SMOTE). SMOTE was applied only within the training portion of each stratified CV split, while the held-out validation folds retained the original class distribution. Variable importance was used to identify the top influential features for each model.

### Statistical analysis

Statistical analyzes were performed in R (V.4.1.2). Machine learning analyzes were conducted using the caret, randomForest, pROC, and themis packages. Categorical variables, including the proportion of subjects with ≥20% donor-derived virome, were compared across the three groups using the Fisher‒Freeman‒Halton exact test, followed by pairwise Fisher's exact tests with Bonferroni correction for multiple comparisons if the overall test was significant. For longitudinal continuous variables, non-parametric repeated measures ANOVA was used to test group, time, and interaction effects. Metrics absent at baseline (e.g., donor-derived virome proportions) were analyzed using only post-FMT time points (weeks 4, 16, 24), whereas intrinsic ecological metrics (Shannon, Chao1) included baseline. Significant interactions were followed by Kruskal‒Wallis tests (between-group) or Friedman tests (within-group), with post hoc Mann‒Whitney U or Wilcoxon signed-rank tests as appropriate. All post hoc *p*-values were Bonferroni-corrected. Alpha diversity indices were calculated using the vegan package. Beta diversity was assessed using Bray-Curtis dissimilarity, and principal coordinates analysis (PCoA) was performed using the phyloseq package. Microbial relative abundance data were transformed using CLR transformation prior to downstream analyzes. Differential abundance analysis of viral contigs was performed using Multivariate Association with linear models (MaAsLin2). Viral contigs with a mean relative abundance of ≥0.01% combined with a prevalence of ≥5% were selected for MaAsLin2 analysis. Trans-kingdom correlations between viral contigs and bacterial species at week 24 were assessed using Spearman's rank correlation based on CLR-transformed data. Correlation networks were constructed using significant associations defined by |r| > 0.5 and *p* < 0.05. Hub nodes were identified based on node degree centrality. A two-sided *p-*value < 0.05 was considered statistically significant.

## Results

A total of 61 subjects were randomly assigned to receive FMT with LSI, FMT alone, or sham with LSI. Among these, 52 subjects had paired VLP-enriched and bulk-DNA metagenomic data available at baseline and week 24, including 17 in the FMT with LSI group, 17 in the FMT alone group, and 18 in the sham with LSI group (Supplementary Figure 1). Baseline characteristics are summarized in Supplementary Table S1. No significant differences were observed between participants included in (*n* = 52) and excluded from (*n* = 9) the virome analysis (Supplementary Table S2). Of the 34 subjects who received FMT, 32 had longitudinal LDL measurements and were included in the RF model analysis. VLP-enriched sequencing and assembly yielded a representative set of 2588 viral contigs after quality filtering and dereplication. Among taxonomically classified reads in the VLP libraries, viral reads constituted the largest fraction overall (median 68.3%), followed by bacterial reads (median 29.5%), whereas fungal and archaeal reads were minimal (maximum 2.99% and 0.07%, respectively) and human-derived reads were low (median 0.08%). A median of 86.7% of VLP reads were recruited to the representative viral contigs catalog, indicating that the catalog captured a substantial proportion of the sequenced viral community in most samples. At the class level, *Malgrandaviricetes* comprised the largest proportion (50.3%), followed by *Arfiviricetes* (10.4%), *Repensiviricetes* (6.9%), and *Caudoviricetes* (5.1%), while 26.2% of contigs remained unclassified (Supplementary Figure 2). In contrast, *Malgrandaviricetes* contigs had a median relative abundance of only 0.7%, whereas *Caudoviricetes* accounted for 12.1% and were detected in all samples (Supplementary Figure 2). Comprehensive annotations and quality metrics of the contigs are provided in Supplementary File 1.

### Post-FMT gut virome changes are associated with LDL response

Post-FMT remodeling of both the gut bacteriome and virome was associated with LDL response. Among the 32 FMT recipients (23 non-responders, 9 responders), changes in lipid-lowering and antidiabetic medications were examined as potential confounders and did not differ significantly between responders and non-responders (Supplementary Table S3). We identified 8 stable virome features and 40 stable bacteriome features through RFE with 50 repeats (selection frequency ≥50%). The virome-based RF classifier achieved a cross-validated accuracy of 84.8% (Kappa = 0.539, AUC = 0.916), while the bacteriome-based RF classifier achieved an accuracy of 81.4% (Kappa = 0.318, AUC = 0.899) (Figure 1). The virome model showed numerically higher balanced accuracy and comparable or higher sensitivity than the bacteriome model across the standard, feature-number-matched (Supplementary Figure 3), and SMOTE sensitivity analyzes (balanced accuracy: 0.756 vs. 0.667 in the standard analysis; 0.734 vs. 0.700 with SMOTE), while overall accuracy and AUC were broadly comparable (Supplementary Table S4). Feature importance analysis identified *Caudoviricetes* contig 278491 as the highest-ranked feature in the virome model (Figure 1a), while *Bulleidia extructa* was the highest-ranked feature in the bacteriome model (Figure 1b).

**Figure 1.**
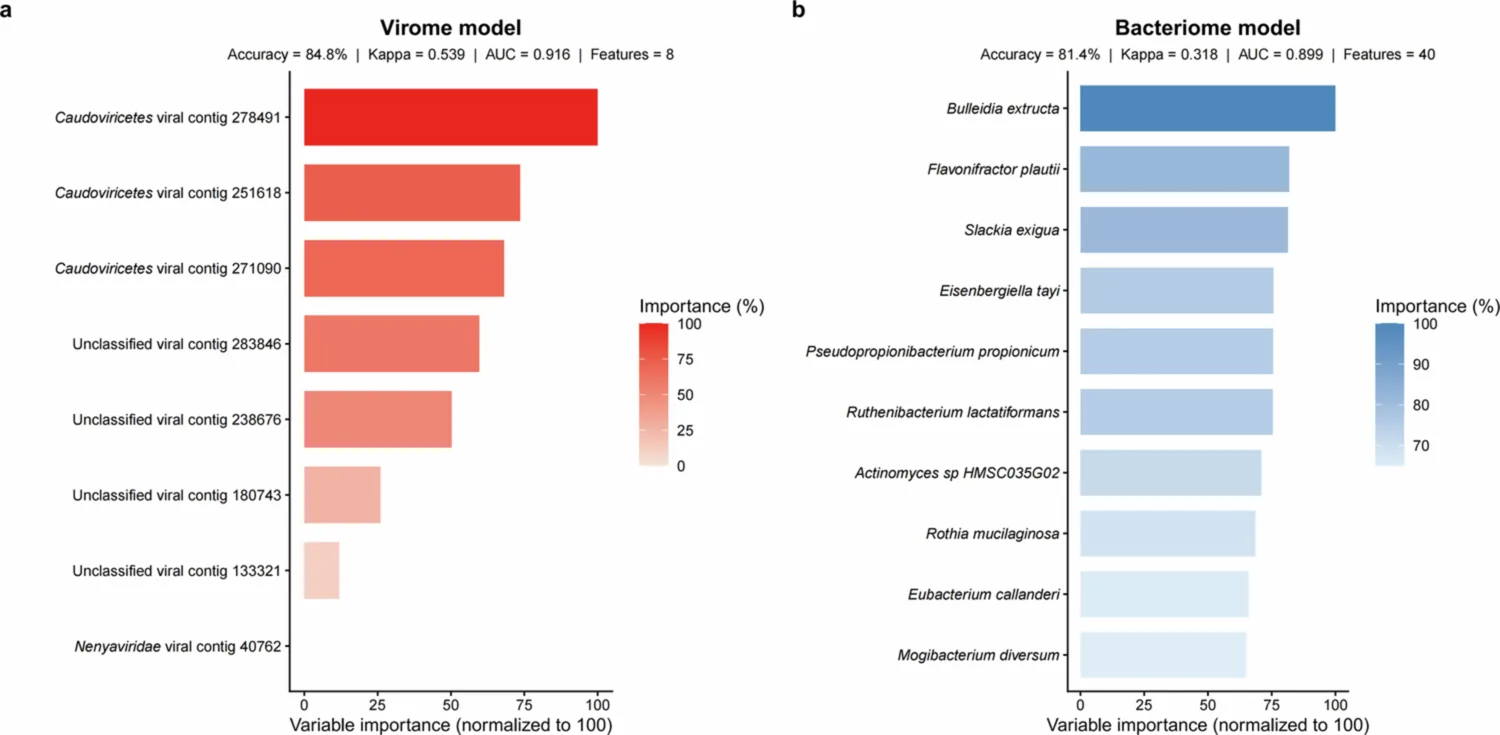
Post-FMT gut virome changes are associated with LDL response. Random forest classifiers were trained on post-FMT changes in gut virome and bacteriome for LDL-response classification among 32 FMT recipients. (a) Stable features in the virome model. (b) Top 10 features in the bacteriome model. Model performance metrics (accuracy, Kappa, AUC) and the number of stable features are shown for both classifiers.

### FMT is associated with sustained engraftment of donor-derived viral contigs independent of LSI

We next examined the engraftment of donor-derived viral contigs and changes in virome composition following FMT. At baseline, four viral contigs were enriched in donors and six viral contigs were enriched in recipients (MaAsLin2, *p* < 0.05; Figure 2a). Three of the four donor-enriched viral contigs and two of the six recipient-enriched viral contigs were classified as *Caudoviricetes*, whereas the remaining contigs were unclassified (Supplementary File 1). Notably, 64.7% of participants in the FMT alone group and 58.8% in the FMT with LSI group reached the 20% threshold, compared with only 5.6% in the sham with LSI group (overall *p* = 0.0005, Fisher‒Freeman‒Halton exact test). Post hoc pairwise comparisons with Bonferroni correction showed that both the FMT alone group (adjusted *p* = 0.0009) and the FMT with LSI group (adjusted *p* = 0.0028) had significantly higher proportions than the sham with LSI group, whereas no significant difference was observed between the two FMT groups (Figure 2b). Both FMT-treated groups had significantly higher proportions than the sham with LSI group at weeks 4, 16, and 24 (all adjusted *p* < 0.05; Figure 2c). No significant differences were observed between the two FMT groups at any time point (all adjusted *p* > 0.05) (Figure 2c). At week 24, donor-derived viral contigs accounted for a median of 21.9% of all detected viral contigs in the FMT alone group, followed by 21.3% in the FMT with LSI group and 7.1% in the sham with LSI group (Figure 2c). Within the FMT with LSI group, no significant difference was detected at week 24 between participants with fair-to-high (*n* = 7) and low (*n* = 10) compliance with LSI (Wilcoxon rank-sum test, *p* = 0.962; Supplementary Figure 4).

**Figure 2.**
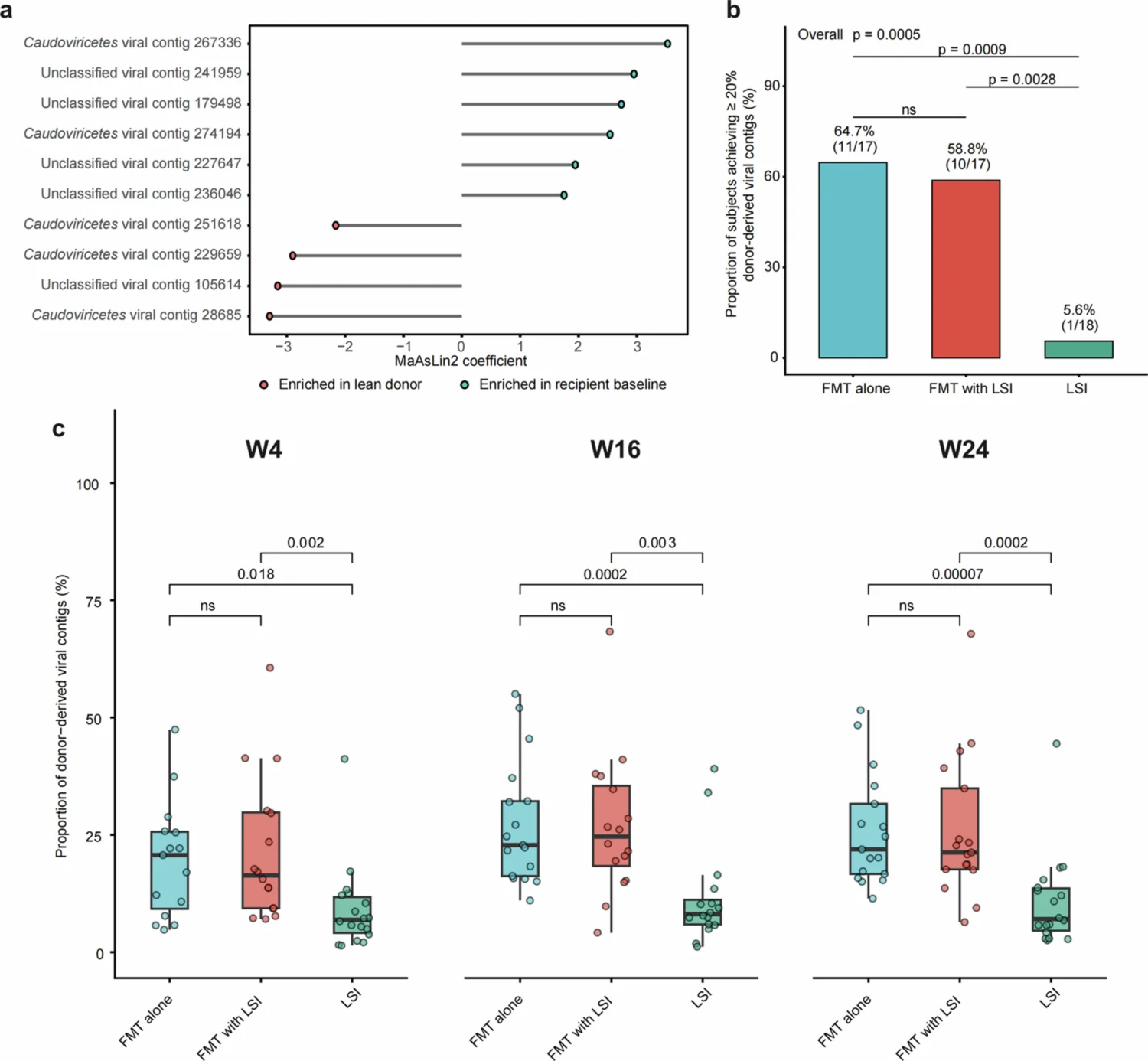
FMT is associated with sustained engraftment of donor-derived viral contigs. (a) Baseline viral contigs enriched in donors vs. recipients with obesity and T2DM (MaAsLin2, *p *< 0.05). (b) Proportion of subjects achieving ≥20% donor-derived viral contigs. Fisher‒Freeman‒Halton exact test (overall *p *= 0.0005) followed by pairwise Fisher's exact tests with Bonferroni correction. (c) Proportions of donor-derived viral contigs at weeks 4, 16, and 24. Stratified between-group comparisons with Bonferroni correction were performed at each time point using Kruskal–Wallis tests followed by the Mann‒Whitney U tests.

### Gut virome alpha diversity and compositional dynamics after FMT

Among the 46 participants with virome data available at all four time points, aligned rank transform analysis for repeated measures revealed a significant effect of time on Shannon diversity (*p* = 0.0009), whereas neither the group effect (*p* = 0.270) nor the group × time interaction (*p* = 0.261) was significant (Figure 3a). For Chao1 richness, the group × time interaction was significant (aligned rank transform analysis, *p* = 0.041), with a between-group difference detected at week 16 (Kruskal‒Wallis, *p* = 0.016). Post hoc comparisons showed that the FMT with LSI group had significantly higher Chao1 richness than the sham with LSI group (adjusted *p* = 0.024), whereas no other significant pairwise differences were observed (all adjusted *p* > 0.05) (Figure 3b). Within-group comparisons showed that both Shannon diversity and Chao1 richness were significantly increased in the FMT alone group at week 24, compared with baseline (both *p* = 0.003), whereas no significant changes were observed in other groups (Supplementary Figures 5 and 6). No significant temporal changes in beta diversity based on Bray‒Curtis dissimilarity to donors were detected in any group by Friedman tests, although PCoA suggested some compositional shifts towards the donors (Figure 3c and d).

**Figure 3.**
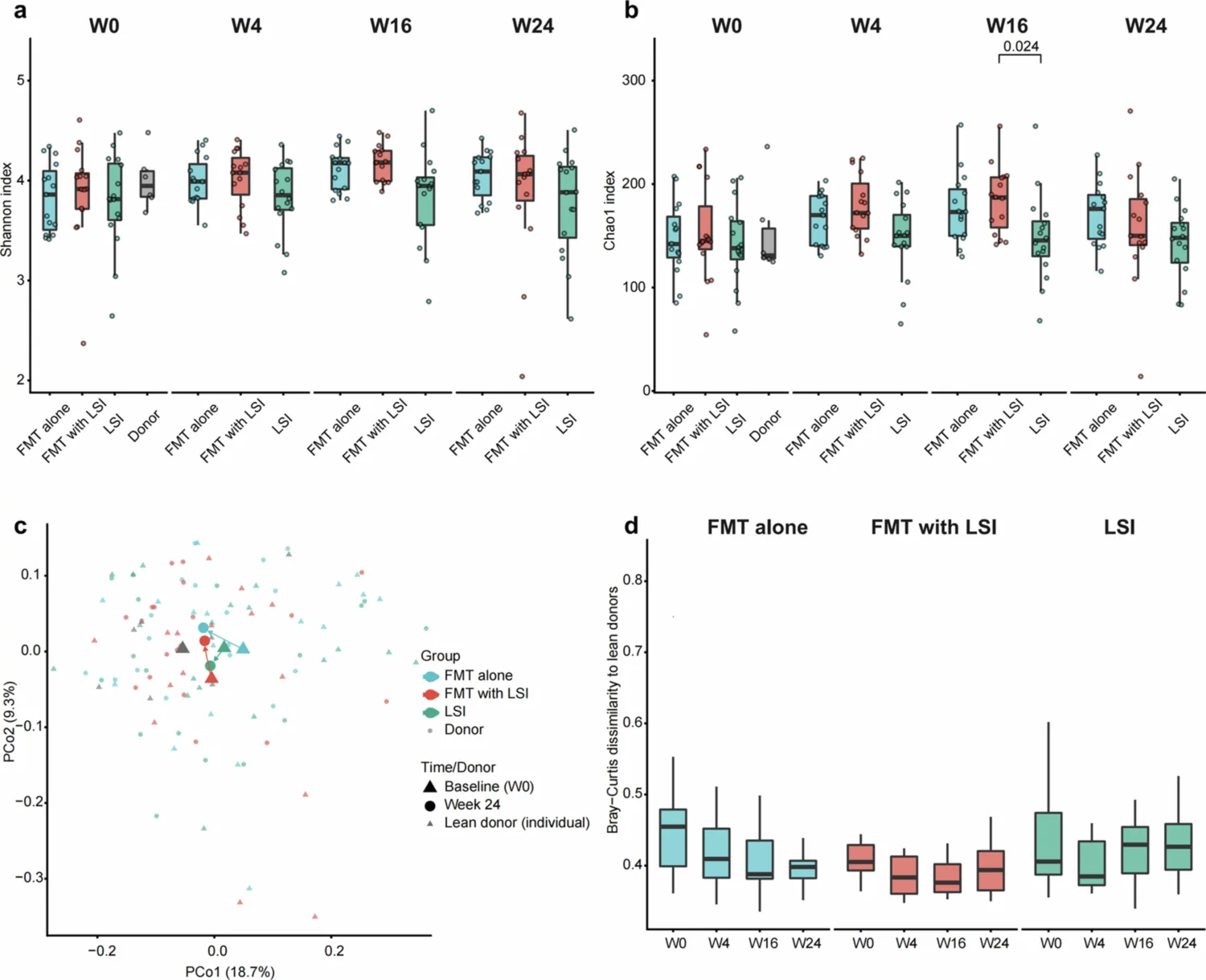
Gut virome alpha diversity and compositional dynamics after FMT. (a) Shannon diversity. Non-parametric repeated measures ANOVA revealed a significant effect of time (*p *= 0.0009), whereas neither the group effect (*p *= 0.270) nor the group × time interaction (*p *= 0.261) was significant. (b) Chao1 richness. Non-parametric repeated measures ANOVA identified a significant group × time interaction (*p *= 0.041). Stratified between-group comparisons showed a significant difference among groups at week 16 (Kruskal‒Wallis test, *p *= 0.016), with higher Chao1 richness in the FMT with LSI group than in the sham with LSI group (Bonferroni-adjusted *p *= 0.024); all other pairwise comparisons were not significant (adjusted *p *> 0.05). (c) Principal coordinates analysis (PCoA) of virome composition across treatment groups and time points. (d) Bray‒Curtis dissimilarity to lean donors.

At week 24, 62, 38, and 3 viral contigs were significantly altered compared with baseline in the FMT alone, FMT with LSI, and sham with LSI groups, respectively (MaAsLin2, *p* < 0.05; Supplementary Figures 7–9), comprising 19, 18, and 2 enriched and 43, 20, and 1 depleted contigs. Most altered contigs in the two FMT-treated groups were classified as *Caudoviricetes*. A total of 10 contigs were enriched in both FMT groups, of which 2 were predicted to infect *Roseburia* and *Mediterraneibacter*. Conversely, 15 viral contigs were depleted in both FMT groups, of which 2 were predicted to infect *Bifidobacterium* species (Figure 4a and b; Supplementary Tables S5 and S6).

**Figure 4.**
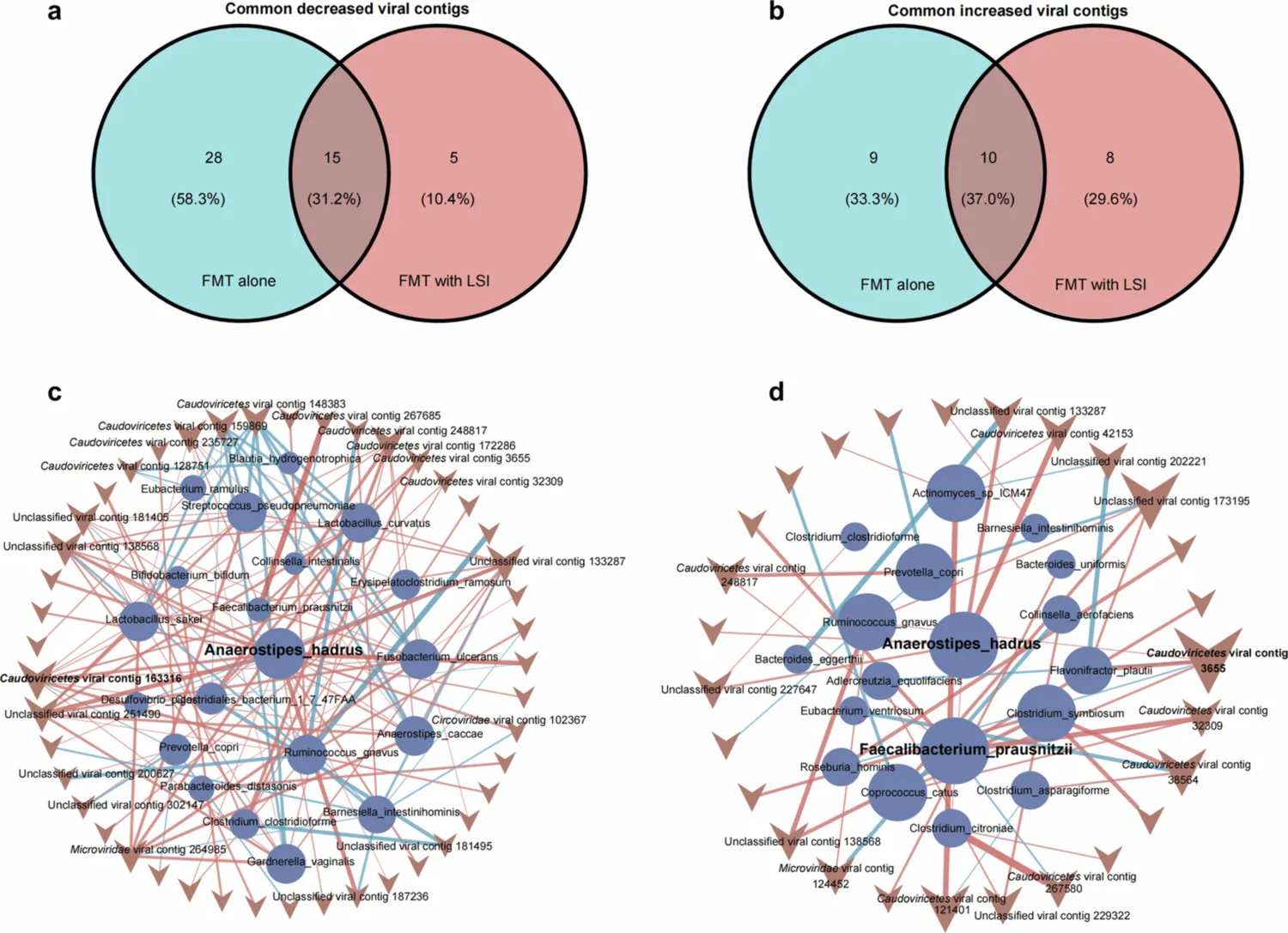
Coordinated remodeling of FMT-responsive viral contigs and virus-bacteria association networks following FMT. (a) Venn diagram showing the overlap of viral contigs significantly depleted at week 24 compared with baseline in the FMT alone and FMT with LSI groups (MaAsLin2, *p *< 0.05). (b) Venn diagram showing the overlap of viral contigs significantly enriched at week 24 compared with baseline in the FMT alone and FMT with LSI groups (MaAsLin2, *p *< 0.05). (c, d) Virus-bacteria association networks in the FMT alone group (c) and the FMT with LSI group (d). Each node represents a microbial taxon. Circles represent bacterial species, and inverted triangles represent viral contigs. Lines (edges) represent significant Spearman correlations between taxa. Red lines indicate positive correlations, and blue lines indicate negative correlations (|r| > 0.5, *p *< 0.05). Node size (degree) reflects the number of direct correlations for a given microbe, with larger nodes representing more highly connected hub taxa.

### Trans-kingdom association networks following FMT

We next assessed the trans-kingdom virus–bacteria associations following FMT. In both FMT groups, viral hubs were predominantly classified as *Caudoviricetes*, with *Anaerostipes hadrus* and *Faecalibacterium prausnitzii* as shared bacterial hubs. The FMT alone group showed additional bacterial hubs including *Anaerostipes caccae, Fusobacterium ulcerans, Lactobacillus* spp., and *Ruminococcus gnavus* (Figure 4c). Several FMT-responsive *Caudoviricetes* contigs were associated with multiple FMT-responsive bacterial species (all |r| > 0.5, *p* < 0.05). For example, *Caudoviricetes* contig 148383, predicted to infect the butyrate-producing genus *Roseburia*, was inversely correlated with several FMT-responsive bacterial taxa, including *Anaerostipes caccae* and *Lactobacillus* spp.

The network was sparser in the FMT with LSI group (Figure 4d). *Caudoviricetes* contig 3655 was the major viral hub. It was positively correlated with *Ruminococcus gnavus*, *Coprococcus catus*, and *Flavonifractor plautii*, and inversely correlated with *Roseburia hominis* and *Adlercreutzia equolifaciens*. Notably, *Faecalibacterium prausnitzii* was positively associated with multiple *Caudoviricetes* contigs, placing this beneficial commensal at a central position in the network following FMT and LSI.

### Specific viral contigs associated with clinical response to FMT with LSI

Finally, we explored viral contigs associated with clinical response in the FMT with LSI group, as subjects in this group exhibited the most pronounced improvements in serum LDL cholesterol and liver CAP score.^8^ For LDL response, subjects were classified as responders (≥0.5 mmol/L reduction, *n* = 8) or non-responders (*n* = 9). At baseline, five viral contigs were enriched in responders and two in non-responders (MaAsLin2, *p* < 0.05; Supplementary Figure 10). Of these, one responder-enriched *Caudoviricetes* contig 288490 remained enriched at week 24 and was predicted to infect *Escherichia*. For liver CAP response, subjects were classified as responders (≥30 dB/m CAP reduction, *n* = 8) and non-responders (*n* = 9). At baseline, five viral contigs were enriched in responders and five in non-responders (MaAsLin2, all *p* < 0.05; Supplementary Figure 10). Several responder-enriched contigs were predicted to infect bacterial genera previously implicated in gut metabolic homeostasis, including *Roseburia* and *Blautia*, whereas one baseline non-responder-enriched *Caudoviricetes* contig 5905 was predicted to infect *Enterococcus* (Supplementary File 1).

## Discussion

Using VLP-enriched metagenomic sequencing, this study provides a longitudinal characterization of gut virome dynamics following FMT in individuals with obesity and T2DM. We found that FMT was associated with sustained changes in virome composition, engraftment of donor-derived viral contigs, virus–bacteria association networks, and virome features associated with metabolic response. Together, these findings extend current evidence that the gut virome is a dynamic component of the intestinal microbial ecosystem following FMT and support its potential relevance to microbiome restructuring and metabolic response.

Independent analyzes of post-FMT virome and bacteriome remodeling showed that both microbial components were associated with metabolic response, with virome features also contributing to LDL response classification, consistent with the close ecological links between viral and bacterial communities.^35^ ^,^ ^36^ Importantly, the virome signals observed here were supported by complementary sequencing and quantification approaches. VLP enrichment resulted in viral reads forming the largest fraction of taxonomically classified reads (median 68.3%), compared with bacterial reads (median 29.5%), while human, fungal, and archaeal reads remained minimal. To minimize potential bias from Phi29 amplification, VLP sequencing was used for viral contig discovery, whereas relative abundance was independently quantified using unamplified bulk metagenomic reads. At the class level, *Malgrandaviricetes* accounted for 50.3% of representative contigs but had a median relative abundance of only 0.7%, whereas *Caudoviricetes* represented only 5.1% of contigs but had a median relative abundance of 12.1%. This discrepancy highlights that contig representation does not necessarily reflect ecological abundance.

Donor-derived viral contig engraftment was detected following FMT and persisted through the 24-week follow-up. We previously reported engraftment of donor-derived bacteriophages following FMT in patients with *Clostridioides difficile* infection (CDI).^37^ The current findings extend this phenomenon to obesity, suggesting that viral engraftment following FMT may occur across distinct disease settings. Engraftment was comparable between the FMT groups, with no differences observed according to LSI compliance. This pattern mirrors the bacterial engraftment previously reported in the same cohort,^8^ suggesting that viral and bacterial engraftment may follow similar dynamics. Although only the FMT alone group showed a significant within-group increase in virome alpha diversity, this should not be interpreted as attenuation of virome remodeling by LSI, given the small sample size and comparable donor-derived contigs between the FMT groups. Virome alpha diversity is highly heterogeneous across disease settings and may not represent a robust ecological signature, whereas changes in viral community composition and specific viral taxa are more consistent indicators of microbiome perturbation.^38^ Thus, donor-derived contigs engraftment, global virome diversity, and clinical improvement may capture distinct aspects of post-FMT ecological remodeling.

*Caudoviricetes* emerged as a prominent feature of post-FMT virome remodeling. Despite comprising only a small proportion of the viral catalog, this class was consistently identified among FMT-responsive contigs, virus‒bacteria association networks, and contigs associated with LDL and liver CAP responses. *Caudoviricetes* are abundant members of the human gut virome^39^ and infect diverse bacterial hosts, including genera associated with metabolic health, such as *Prevotella*,^40^ ^,^ ^41^ *Faecalibacterium*, ^42^ and *Roseburia*.^43^ Alterations in *Caudovirales*-dominated phage communities have been reported in obesity and metabolic syndrome,^44^ and recent multicohort evidence showed increased abundance of *Caudoviricetes* in individuals with obesity.^45^ This observation is also consistent with our previous study showing enrichment of *Caudovirales* following FMT in patients with CDI.^37^

In line with this, predicted hosts of FMT-responsive *Caudoviricetes* contigs included members of the *Lachnospiraceae* family, such as *Roseburia* and *Mediterraneibacter*, as well as *Bifidobacterium* species. Trans-kingdom analyzes further linked these contigs with metabolically relevant bacteria, including *Anaerostipes hadrus*, *Faecalibacterium prausnitzii*, and *Lactobacillus* species, which have been associated with butyrate production, intestinal barrier integrity, insulin sensitivity, anti-inflammatory properties, and bile acid metabolism.^46-51^ Together with the association of several *Caudoviricetes* contigs with LDL and liver CAP responses, these findings highlight the potential relevance of *Caudoviricetes* to post-FMT virome remodeling in obesity, although their functional contribution to metabolic response requires mechanistic validation.

This study has several strengths, including its randomized, double-blind design, longitudinal follow-up, and integration of VLP-enriched and bulk metagenomic sequencing. However, several limitations should be acknowledged. First, the moderate sample size and the observational nature limit causal interpretation. Second, although Phi29-mediated amplification was used only for viral contig discovery, its preferential amplification of certain viral genomes, particularly small circular ssDNA viruses, may have biased contig recovery.^44^ Third, our analysis was restricted to DNA viruses and did not capture RNA viruses. Fourth, residual bacterial DNA cannot be entirely excluded despite decontamination. Finally, mechanistic studies are needed to establish how specific virus‒bacteria interactions influence metabolic responses to FMT.

In conclusion, our findings highlight the gut virome as an underappreciated component of post-FMT microbial remodeling in obesity and T2DM. Sustained donor-derived viral engraftment, virus‒bacteria associations, and virome features associated with clinical response support the relevance of the gut virome to FMT outcomes. These findings highlight an integrated virus‒bacteria‒host framework for understanding FMT and provide a foundation for future mechanistic studies.

## Supplementary Material

Supplementary File 1_revised.xlsx Supplementary File 1_revised.xlsx

Supplementary Table S1_revised.docx Supplementary Table S1_revised.docx

Supplementary Table S2_revised.docx Supplementary Table S2_revised.docx

Supplementary Table S6_revised.csv Supplementary Table S6_revised.csv

Supplementary File 2_revised.pdf Supplementary File 2_revised.pdf

Supplementary Figures and Legends_revised.pdf Supplementary Figures and Legends_revised.pdf

Supplementary Table S5_revised.csv Supplementary Table S5_revised.csv

Supplementary Table S4_revised.docx Supplementary Table S4_revised.docx

Supplementary Material Supplementary Table S3_revised.docx

## Data Availability

The sequencing data of this article is available in the NCBI under Bioproject, PRJNA644456. All data and key analysis code needed to evaluate the findings are presented in the paper and/or the Supplementary Files 1 and 2.

## Abbreviations

ObT2: obese subjects with type 2 diabetes
FMT: fecal microbiota transplantation
VLP: virus-like particle
LSI: lifestyle intervention
LDL: low-density lipoprotein
CAP: controlled attenuation parameter
T2DM: type 2 diabetes mellitus
BMI: body mass index
ANI: average nucleotide identity
AF: alignment fraction
RPKM: reads per kilobase million
CLR: centered log-ratio
PCoA: principal coordinates analysis
FVT: fecal virome transplantation
RF: Random Forest
RFE: Recursive feature elimination
CV: Cross-validation
AUC: Area under the receiver operating characteristic curve
SMOTE: Synthetic Minority Over-sampling Technique

## References

[1] Ley RE , Bäckhed F , Turnbaugh P , Lozupone CA , Knight RD , Gordon JI . Obesity alters gut microbial ecology. Proc Natl Acad Sci U S A. 2005;102(31):11070–11075. doi: 10.1073/pnas.0504978102. PMC1176910 16033867

[2] Xu Z , Jiang W , Huang W , Lin Y , Chan FKL , Ng SC . Gut microbiota in patients with obesity and metabolic disorders - a systematic review. Genes Nutr. 2022;17(1):2. doi: 10.1186/s12263-021-00703-6. PMC8903526 35093025

[3] de la Cuesta-Zuluaga J , Huus KE , Youngblut ND , Escobar JS , Ley RE . Obesity is the main driver of altered gut microbiome functions in the metabolically unhealthy. Gut Microbes. 2023;15(2):2246634. doi: 10.1080/19490976.2023.2246634. PMC10486298 37680093

[4] Zhang Y , Liu R , Chen Y , Cao Z , Liu C , Bao R , Wang Y , Huang S , Pan S , Qin L , et al. Akkermansia muciniphila supplementation in patients with Overweight/Obese type 2 diabetes: efficacy depends on its baseline levels in the gut. Cell Metab. 2025;37(3):592–605e6. doi: 10.1016/j.cmet.2024.12.010. 39879980

[5] Wang H , Tian J , Mi J . Clinical effectiveness of fecal microbial transplantation for metabolic syndrome: advances in clinical efficacy and multi-omics research. Curr Res Microb Sci. 2025;9:100415. doi: 10.1016/j.crmicr.2025.100415. PMC12205572 40583967

[6] Li K , Li Z , Liu L . Long-term efficacy of dapagliflozin combined with bifidobacterium triple viable tablets on metabolic syndrome and gut microbiota in Overweight/Obese patients with type 2 diabetes. Endocr Connect. 2025;14(9):e250219. doi: 10.1530/ec-25-0219. PMC12412283 40827948

[7] Dolatkhah N , Nourizadeh E , Aghamohammadzadeh N , Yahyavi S , Eslamian F , Hashemian M . The promising effects of a multi-species synbiotic preparation on metabolic profile in elderly patients with type 2 diabetes and high cardiovascular risk: a randomized, triple-blind, placebo-controlled trial. Nutr Diabetes. 2025;16(1):2. doi: 10.1038/s41387-025-00408-4. PMC12770592 41413231

[8] Ng SC , Xu Z , Mak JWY , Yang K , Liu Q , Zuo T , Tang W , Lau L , Lui RN , Wong SH , et al. Microbiota engraftment after faecal microbiota transplantation in obese subjects with type 2 diabetes: a 24-Week, double-blind, randomised controlled trial. Gut. 2022;71(4):716–723. doi: 10.1136/gutjnl-2020-323617. 33785557

[9] Erez Z , Steinberger-Levy I , Shamir M , Doron S , Stokar-Avihail A , Peleg Y , Melamed S , Leavitt A , Savidor A , Albeck S , et al. Communication between viruses guides lysis-lysogeny decisions. Nature. 2017;541(7638):488–493. doi: 10.1038/nature21049. PMC5378303 28099413

[10] Hsu BB . Towards the characterization and engineering of bacteriophages in the gut microbiome. mSystems. 2021;6(4):e0073521. doi: 10.1128/mSystems.00735-21. PMC8407375 34427526

[11] Deng Y , Jiang S , Duan H , Shao H , Duan Y . Bacteriophages and their potential for treatment of metabolic diseases. J Diabetes. 2024;16(11):e70024. doi: 10.1111/1753-0407.70024. PMC11586638 39582431

[12] Kirk D , Costeira R , Visconti A , Khan Mirzaei M , Deng L , Valdes AM , Menni C . Bacteriophages, gut bacteria, and microbial pathways interplay in cardiometabolic health. Cell Rep. 2024;43(2):113728. doi: 10.1016/j.celrep.2024.113728. PMC11554570 38300802

[13] Chica Cardenas LA , Leonard MM , Baldridge MT , Handley SA . Gut virome dynamics: from commensal to critical player in health and disease. Nat Rev Gastroenterol Hepatol. 2026;23(2):126–144. doi: 10.1038/s41575-025-01134-z. PMC13112531 41193697

[14] Yang K , Niu J , Zuo T , Sun Y , Xu Z , Tang W , Liu Q , Zhang J , Ng EK , Wong SK , et al. Alterations in the gut virome in obesity and type 2 diabetes mellitus. Gastroenterology. 2021;161(4):1257–1269 e13. doi: 10.1053/j.gastro.2021.06.056. 34175280

[15] Lam S , Bai X , Shkoporov AN , Park H , Wu X , Lan P , Zuo T . Roles of the gut virome and mycobiome in faecal microbiota transplantation. Lancet Gastroenterol Hepatol. 2022;7(5):472–484. doi: 10.1016/s2468-1253(21)00303-4. 35276080

[16] Gonzalez Pastor B , Shkoporov AN , Hill C . Not just passengers: phages as agents of genetic exchange in fecal microbiota transplantation. Cell Host Microbe. 2026;34:780–789. doi: 10.1016/j.chom.2026.03.017. 41990750

[17] Draper LA , Ryan FJ , Smith MK , Jalanka J , Mattila E , Arkkila PA , Ross RP , Satokari R , Hill C . Long-term colonisation with donor bacteriophages following successful faecal microbial transplantation. Microbiome. 2018;6(1):220. doi: 10.1186/s40168-018-0598-x. PMC6288847 30526683

[18] Liu Q , Xu Z , Dai M , Su Q , Leung Chan FK , Ng SC . Faecal microbiota transplantations and the role of bacteriophages. Clin Microbiol Infect. 2023;29(6):689–694. doi: 10.1016/j.cmi.2022.11.012. 36414201

[19] Wortelboer K , de Jonge PA , Scheithauer TPM , Attaye I , Kemper EM , Nieuwdorp M , Herrema H . Phage-microbe dynamics after sterile faecal filtrate transplantation in individuals with metabolic syndrome: a double-blind, randomised, placebo-controlled clinical trial assessing efficacy and safety. Nat Commun. 2023;14(1):5600. doi: 10.1038/s41467-023-41329-z. PMC10497675 37699894

[20] Rasmussen TS , Mentzel CMJ , Kot W , Castro-Mejía JL , Zuffa S , Swann JR , Hansen LH , Vogensen FK , Nielsen DS . Faecal virome transplantation decreases symptoms of type 2 diabetes and obesity in a murine model. Gut. 2020;69(12):2122–2130. doi: 10.1136/gutjnl-2019-320005. 32165408

[21] Mao X , Larsen SB , Zachariassen LSF , Brunse A , Adamberg S , Mejia JLC , Nielsen DS , Hansen AK , Rasmussen TS . Transfer of modified gut viromes improves symptoms associated with metabolic syndrome in obese Male mice. Nat Commun. 2024;15(1):4704. doi: 10.1038/s41467-024-49152-w. PMC11148109 38830845

[22] Cervantes-Echeverría M , Jimenez-Rico MA , Manzo R , Hernández-Reyna A , Cornejo-Granados F , Bikel S , González V , Hurtado Ramírez JM , Sánchez-López F , Salazar-León J , et al. Human-derived fecal virome transplantation (Fvt) reshapes the murine gut microbiota and virome, enhancing glucose regulation. PLoS One. 2025;20(12):e0337760. doi: 10.1371/journal.pone.0337760. PMC12680211 41348832

[23] Bolger AM , Lohse M , Usadel B . Trimmomatic: a flexible trimmer for illumina sequence data. Bioinformatics. 2014;30(15):2114–2120. doi: 10.1093/bioinformatics/btu170. PMC4103590 24695404

[24] Langmead B , Salzberg SL . Fast gapped-read alignment with bowtie 2. Nat Methods. 2012;9(4):357–359. doi: 10.1038/nmeth.1923. PMC3322381 22388286

[25] Wood DE , Lu J , Langmead B . Improved metagenomic analysis with kraken 2. Genome Biol. 2019;20(1):257. doi: 10.1186/s13059-019-1891-0. PMC6883579 31779668

[26] Lu J , Breitwieser FP , Thielen P , Salzberg SL . Bracken: estimating species abundance in metagenomics data. PeerJ Comput Sci. 2017;3:e104. doi: 10.7717/peerj-cs.104. PMC12016282 40271438

[27] Li D , Liu CM , Luo R , Sadakane K , Lam TW . Megahit: an ultra-fast single-node solution for large and complex metagenomics assembly via succinct de bruijn graph. Bioinformatics. 2015;31(10):1674–1676. doi: 10.1093/bioinformatics/btv033. 25609793

[28] Nayfach S , Camargo AP , Schulz F , Eloe-Fadrosh E , Roux S , Kyrpides NC . Checkv assesses the quality and completeness of metagenome-assembled viral genomes. Nat Biotechnol. 2021;39(5):578–585. doi: 10.1038/s41587-020-00774-7. PMC8116208 33349699

[29] Camargo AP , Roux S , Schulz F , Babinski M , Xu Y , Hu B , Chain PSG , Nayfach S , Kyrpides NC . Identification of mobile genetic elements with genomad. Nat Biotechnol. 2024;42(8):1303–1312. doi: 10.1038/s41587-023-01953-y. PMC11324519 37735266

[30] Buchfink B , Xie C , Huson DH . Fast and sensitive protein alignment using diamond. Nat Methods. 2015;12(1):59–60. doi: 10.1038/nmeth.3176. 25402007

[31] Huson DH , Beier S , Flade I , Górska A , El-Hadidi M , Mitra S , Ruscheweyh H , Tappu R , Poisot T . Megan community edition - interactive exploration and analysis of large-scale microbiome sequencing data. PLoS Comput Biol. 2016;12(6):e1004957. doi: 10.1371/journal.pcbi.1004957. PMC4915700 27327495

[32] Shang J , Tang X , Sun Y . Phatyp: predicting the lifestyle for bacteriophages using bert. Brief Bioinform. 2023;24(1). doi: 10.1093/bib/bbac487. PMC9851330 36659812

[33] Peng Y , Zhu J , Wang S , Liu Y , Liu X , DeLeon O , Xu Z , Zhang X , Zhao S , Liang S , et al. A metagenome-assembled genome inventory for children reveals early-life gut bacteriome and virome dynamics. Cell Host Microbe. 2024;32(12):2212–2230.e8. doi: 10.1016/j.chom.2024.10.017. 39591974

[34] Aroney STN , Newell RJP , Nissen JN , Camargo AP , Tyson GW , Woodcroft BJ . Coverm: read alignment statistics for metagenomics. Bioinformatics. 2025;41(4), btaf147. 10.1093/bioinformatics/btaf147. PMC11993303 40193404

[35] Shkoporov AN , Turkington CJ , Hill C . Mutualistic interplay between bacteriophages and bacteria in the human gut. Nat Rev Microbiol. 2022;20(12):737–749. doi: 10.1038/s41579-022-00755-4. 35773472

[36] Liang G , Bushman FD . The human virome: assembly, composition and host interactions. Nat Rev Microbiol. 2021;19(8):514–527. doi: 10.1038/s41579-021-00536-5. PMC8008777 33785903

[37] Zuo T , Wong SH , Lam K , Lui R , Cheung K , Tang W , Ching JYL , Chan PKS , Wu JCY , Yu J , et al. Bacteriophage transfer during faecal microbiota transplantation in clostridium difficile infection is associated with treatment outcome. Gut. 2018;67(4):634–643. doi: 10.1136/gutjnl-2017-313952. PMC5868238 28539351

[38] Wheatley RM , Holtappels D , Koskella B . Evaluation of bacteriophages as a signature of microbiome health: a systematic review and meta-analysis. Lancet Microbe. 2025;6(12):101196. doi: 10.1016/j.lanmic.2025.101196. 41077053

[39] Gregory AC , Zablocki O , Zayed AA , Howell A , Bolduc B , Sullivan MB . The gut virome database reveals age-dependent patterns of virome diversity in the human gut. Cell Host Microbe. 2020;28(5):724–740.e8. doi: 10.1016/j.chom.2020.08.003. PMC7443397 32841606

[40] Wu X , Mu B , Li G , Du R , Park S . Gut microbial composition, oxidative stress, and immunity in metabolic disease: toward personalized interventions. Antioxidants (Basel). 2026;15(2):175. doi: 10.3390/antiox15020175. PMC12938183 41750556

[41] Yang C , Lan R , Zhao L , Pu J , Hu D , Yang J , Zhou H , Han L , Ye L , Jin D , et al. Prevotella copri alleviates hyperglycemia and regulates gut microbiota and metabolic profiles in mice. mSystems. 2024;9(7):e0053224. doi: 10.1128/msystems.00532-24. PMC11265406 38934548

[42] Qin J , Li Y , Cai Z , Li S , Zhu J , Zhang F , Liang S , Guan Y , Shen D , Peng Y , et al. A metagenome-wide association study of gut microbiota in type 2 diabetes. Nature. 2012;490(7418):55–60. doi: 10.1038/nature11450. 23023125

[43] Huang W , Zhu W , Lin Y , Chan FKL , Xu Z , Ng SC . Roseburia hominis improves host metabolism in diet-induced obesity. Gut Microbes. 2025;17(1):2467193. doi: 10.1080/19490976.2025.2467193. PMC11845086 39976263

[44] Bikel S , López-Leal G , Cornejo-Granados F , Gallardo-Becerra L , García-López R , Sánchez F , Equihua-Medina E , Ochoa-Romo JP , López-Contreras BE , Canizales-Quinteros S , et al. Gut dsdna virome shows diversity and richness alterations associated with childhood obesity and metabolic syndrome. iSci. 2021;24(8):102900. doi: 10.1016/j.isci.2021.102900. PMC8361208 34409269

[45] Gallardo-Nuell L , Rosell-Díaz M , Garre-Olmo J , Puig J , Ramos R , Pons J , Pérez-Brocal V , Moya A , Mayneris-Perxachs J , Fernández-Real JM . Ecological restructuring of the nonbacterial fecal microbiome in obesity across human cohorts. Gut Microbes. 2026;18(1):2701446. doi: 10.1080/19490976.2026.2701446. PMC13378717 42458961

[46] Miquel S , Martín R , Rossi O , Bermúdez-Humarán LG , Chatel JM , Sokol H , Thomas M , Wells J , Langella P . Faecalibacterium prausnitzii and human intestinal health. Curr Opin Microbiol. 2013;16(3):255–261. doi: 10.1016/j.mib.2013.06.003. 23831042

[47] Liu Y , Kuang W , Li M , Wang Z , Liu Y , Zhao M , Huan H , Yang Y . Cholesterol-lowering mechanism of lactobacillus bile salt hydrolase through regulation of bifidobacterium pseudolongum in the gut microbiota. Nutrients. 2025;17(18):3019. doi: 10.3390/nu17183019. PMC12472961 41010544

[48] Cao J , Song A , Zhang Q , Zhang T , Jia X , Li B , Lan C , Hu Y . Lactobacillus paracasei Lp18 ameliorated inflammation and intestinal barrier dysfunction in severe acute pancreatitis via gut microbiota-mediated regulation of butyrate metabolism. Front Microbiol. 2026;17:1765127. doi: 10.3389/fmicb.2026.1765127. PMC12936515 41767557

[49] Zhang C , Gao Y , Huang Y , Qin Y , Li Y , Chen Q , Wu T , Deng D , Han M , Hayer J . Multi-omics analysis reveals the mechanism of lactobacillus plantarum in alleviating metabolic disorders in type 2 diabetic mice through the gut-liver axis. mSystems. 2025;10(11):e0096525. doi: 10.1128/msystems.00965-25. PMC12625740 41165343

[50] Cui J , Ramesh G , Wu M , Jensen ET , Crago O , Bertoni AG , Gao C , Hoffman KL , Sheridan PA , Wong KE , et al. Butyrate-producing bacteria and insulin homeostasis: the microbiome and insulin longitudinal evaluation study (Miles). Diabetes. 2022;71(11):2438–2446. doi: 10.2337/db22-0168. PMC9630078 35972231

[51] Liu D , Xie LS , Lian S , Li K , Yang Y , Wang WZ , Hu S , He Z , Tamaki H . Anaerostipes hadrus, a butyrate-producing bacterium capable of metabolizing 5-Fluorouracil. mSphere. 2024;9(4):e0081623. doi: 10.1128/msphere.00816-23. PMC11036815 38470044

