# Supplementary material for: Gut virome remodeling after fecal microbiota transplantation is associated with virus–bacteria co-variation and metabolic response in obesity and type 2 diabetes
Source: Gut Microbes. 2026 Oct 23;18(1):2746820. doi: 10.1080/19490976.2026.2746820 (PMC13699845; doi:10.1080/19490976.2026.2746820)
Supplement: Supplementary file 1 [file KGMI_A_2746820_SM0691.pdf]

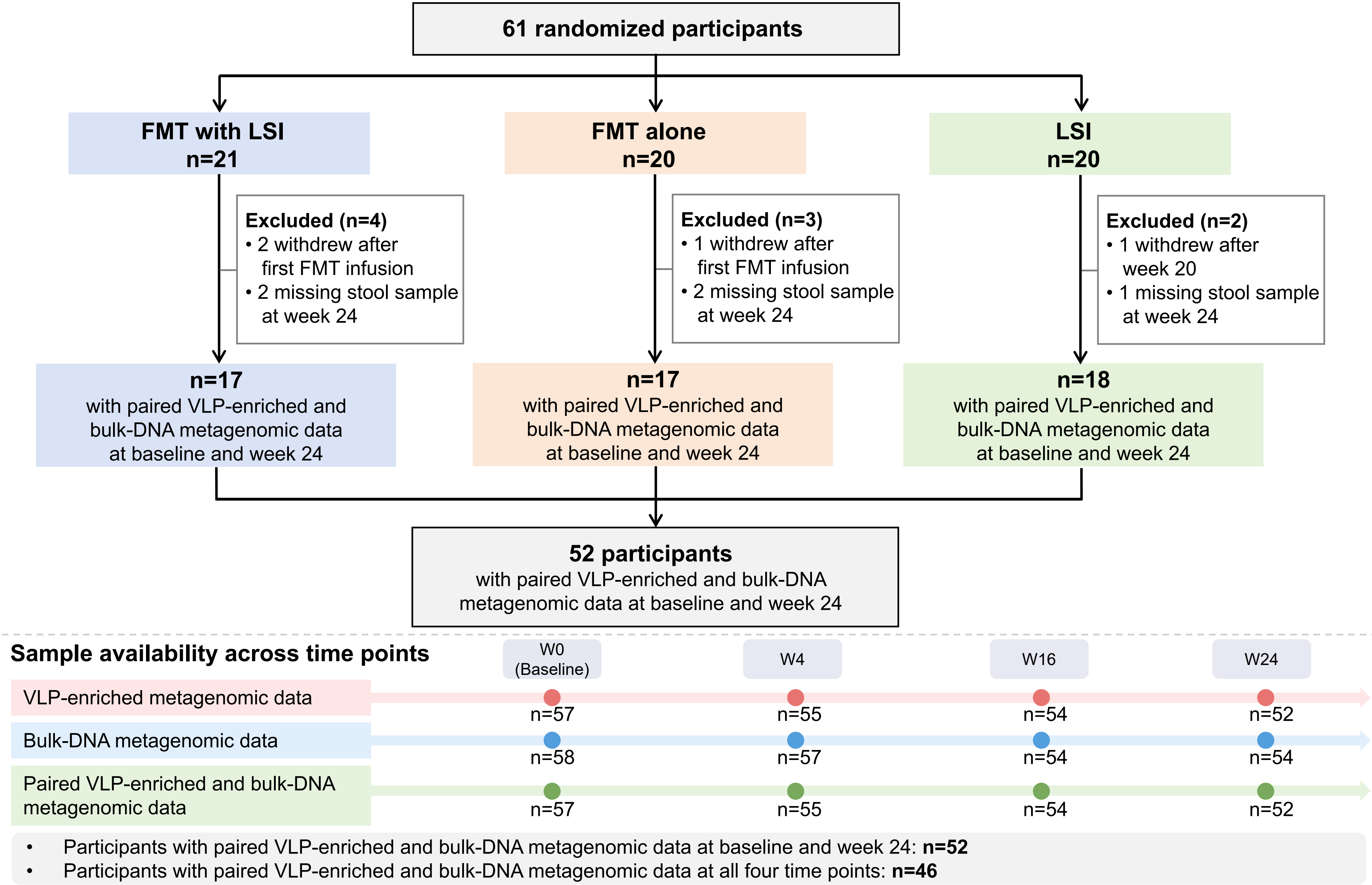

Supplementary Figure 1. Participant flow and sample availability across time points. The figure shows participant allocation, exclusion, and the numbers with available VLP-enriched, bulk-DNA, and paired metagenomic data at each time point. Nine participants were excluded from the paired baseline and week-24 analyses because of study withdrawal (n=4) or missing week-24 stool samples (n=5), with group-specific reasons shown in the figure. Missing VLP-enriched metagenomic data at individual time points were due to VLP libraries that did not pass sequencing quality control.

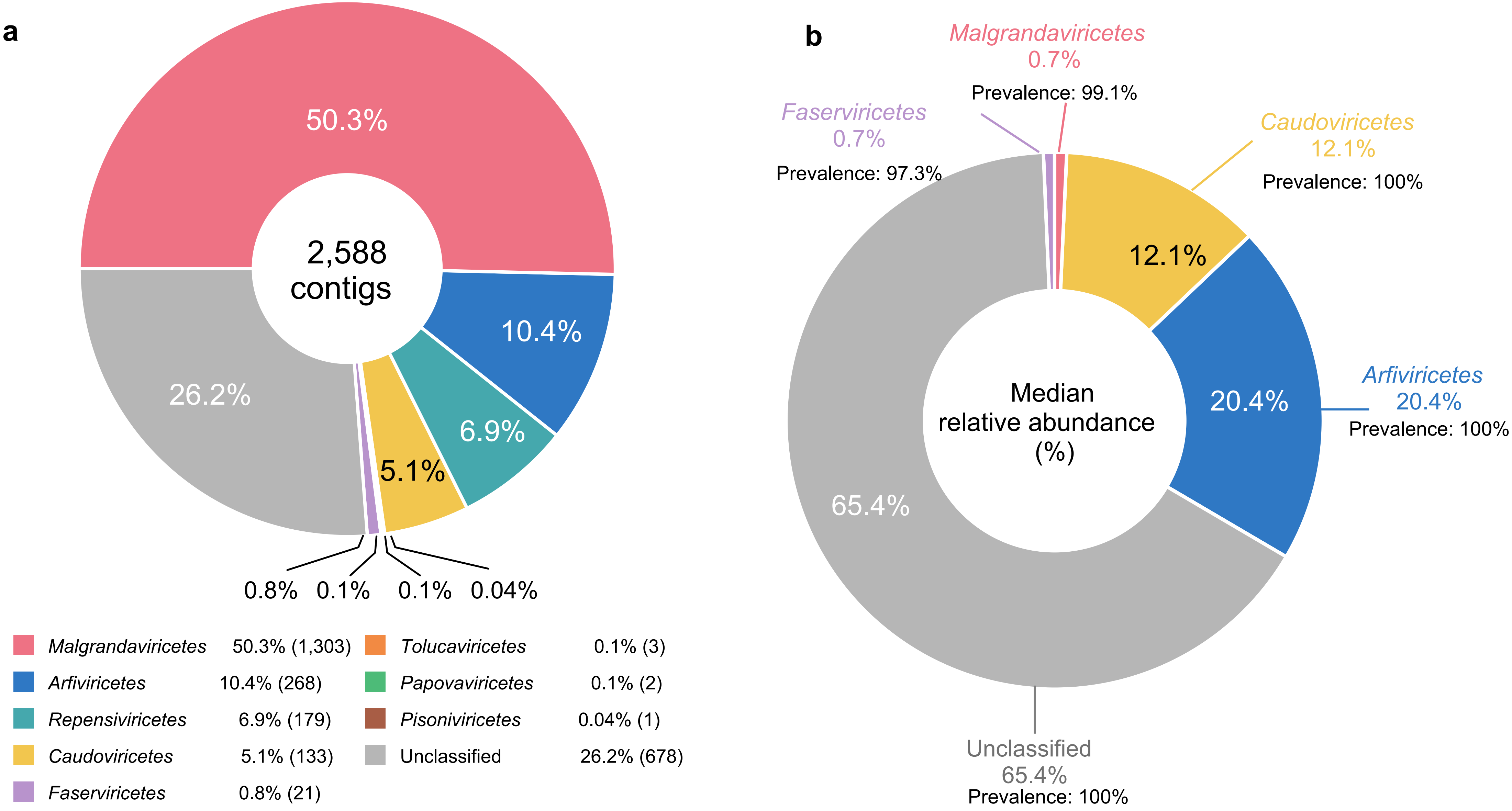

Supplementary Figure 2. Class-level taxonomic composition, relative abundance, and prevalence of the viral contigs. (a) Class-level taxonomic composition of the 2,588 representative viral contigs obtained after quality filtering and dereplication. Percentages represent the proportion of contigs assigned to each viral class. (b) Class-level median relative abundance and prevalence based on bulk metagenomic reads. The five classes with the highest median relative abundance are shown.

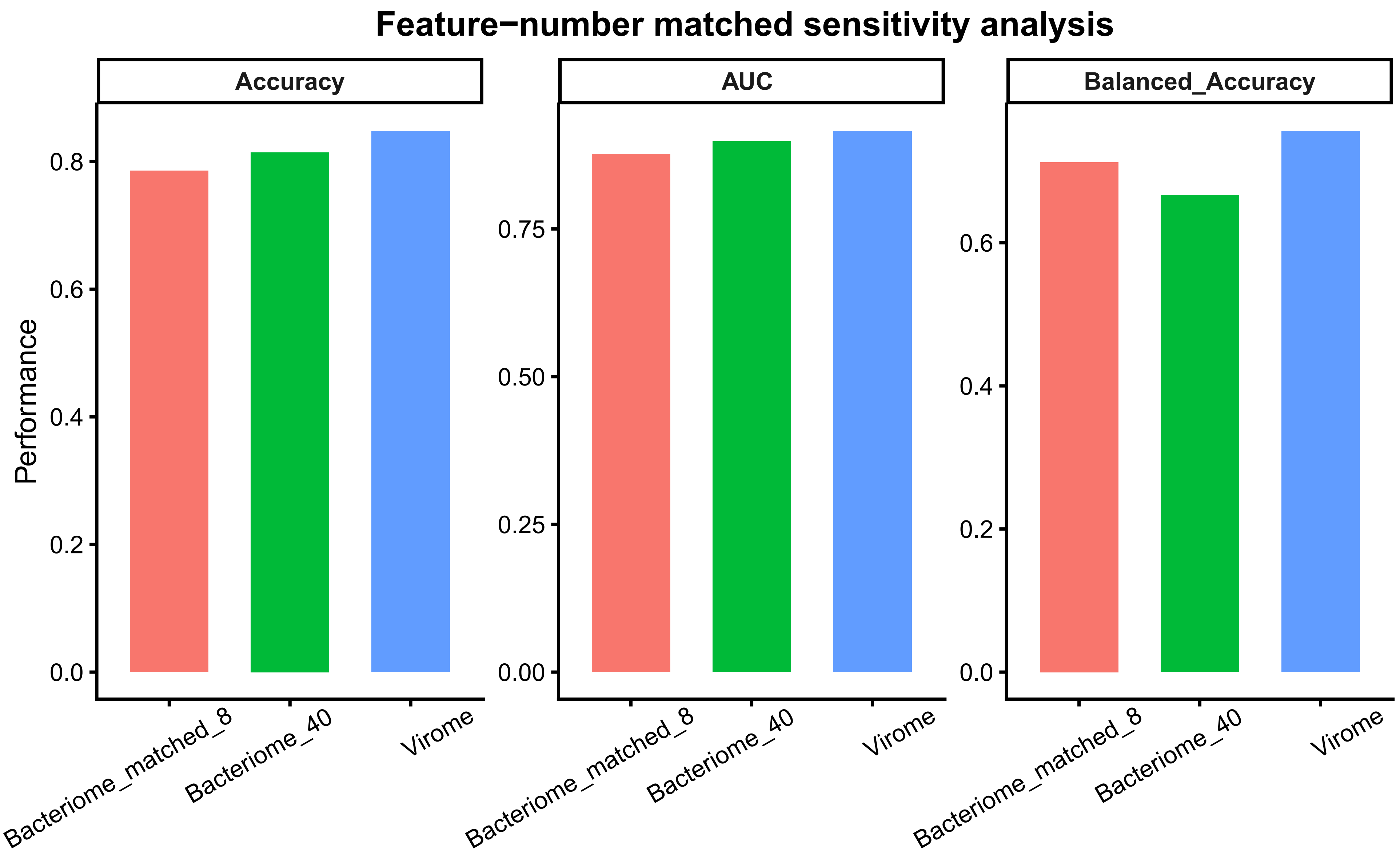

Supplementary Figure 3. Feature-number-matched sensitivity analysis of virome and bacteriome prediction models. Comparison of accuracy, area under the receiver operating characteristic curve (AUC), and balanced accuracy among the virome model (8 stable features), bacteriome model (40 stable features), and feature-number-matched bacteriome model (8 features).

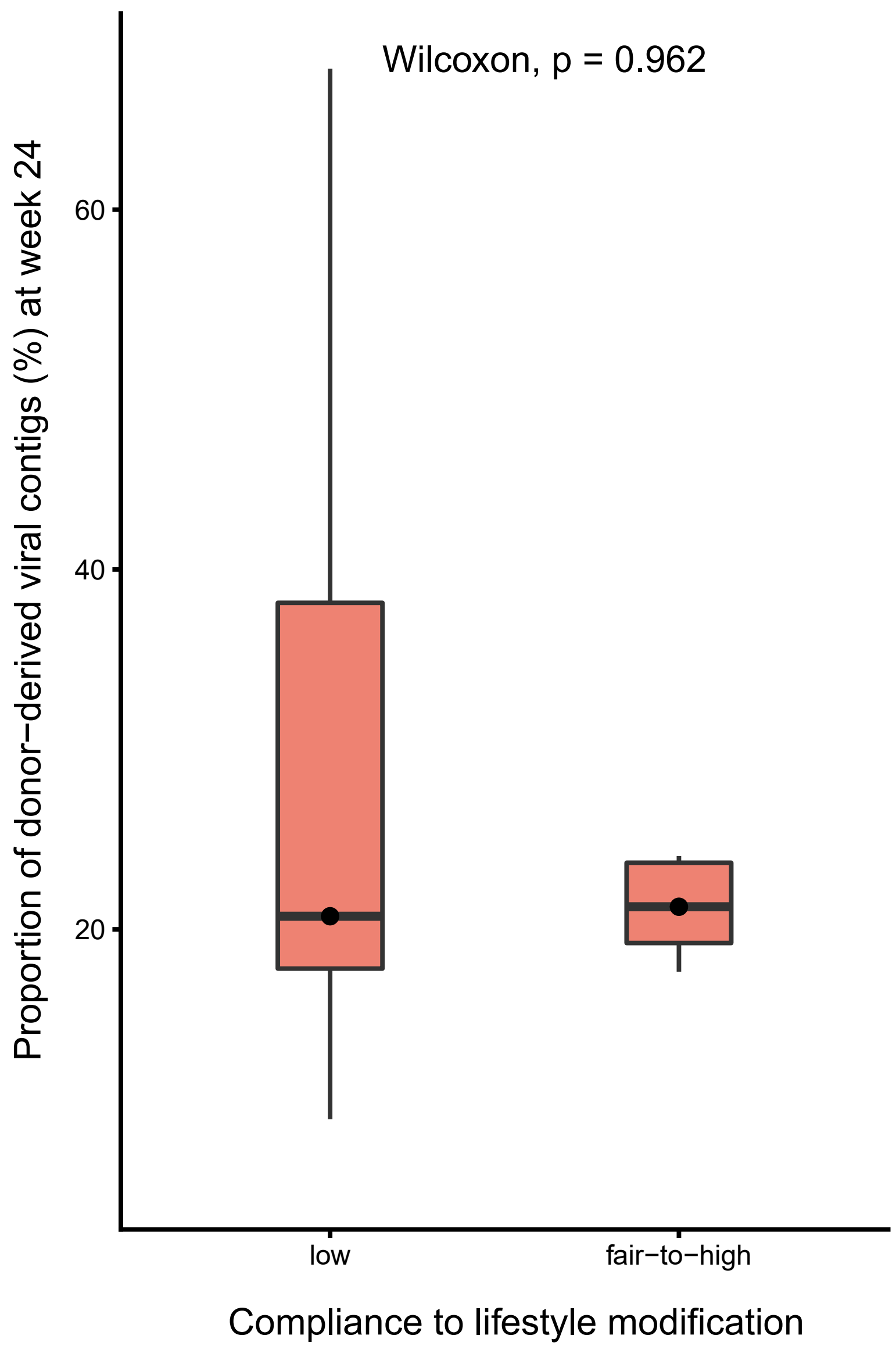

Supplementary Figure 4. Donor-derived virome engraftment in the FMT with LSI group stratified by compliance level. Comparison of fair-to-high (n=7) vs. low (n=10) compliance to LSI in the FMT with LSI group at week 24 (Wilcoxon rank-sum test).

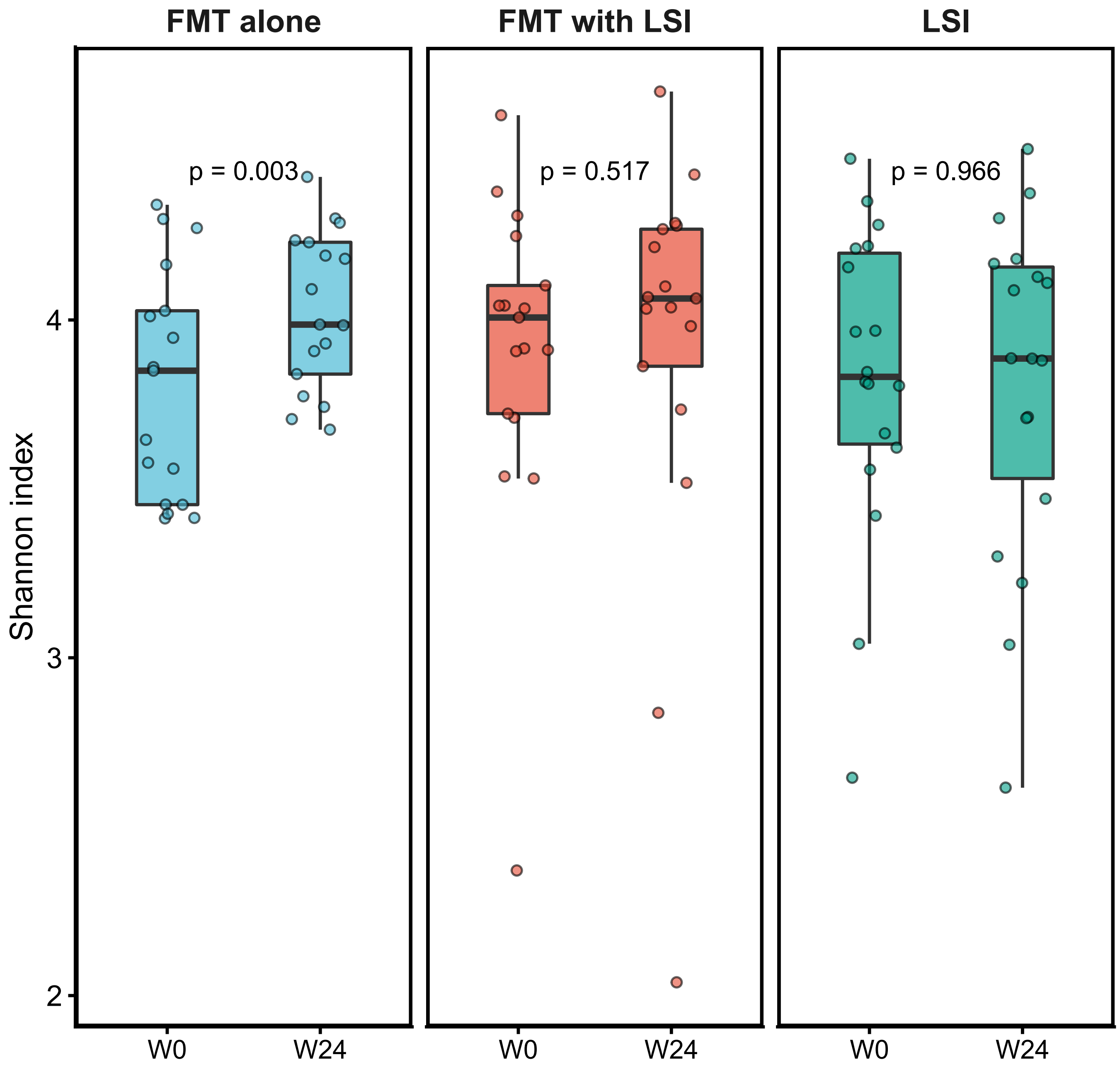

Supplementary Figure 5. Shannon diversity changes at week 24 compared with baseline. Shannon diversity at baseline and week 24 is shown for the FMT alone, FMT with LSI, and sham with LSI groups. Within-group comparisons between baseline and week 24 were performed using paired Wilcoxon tests.

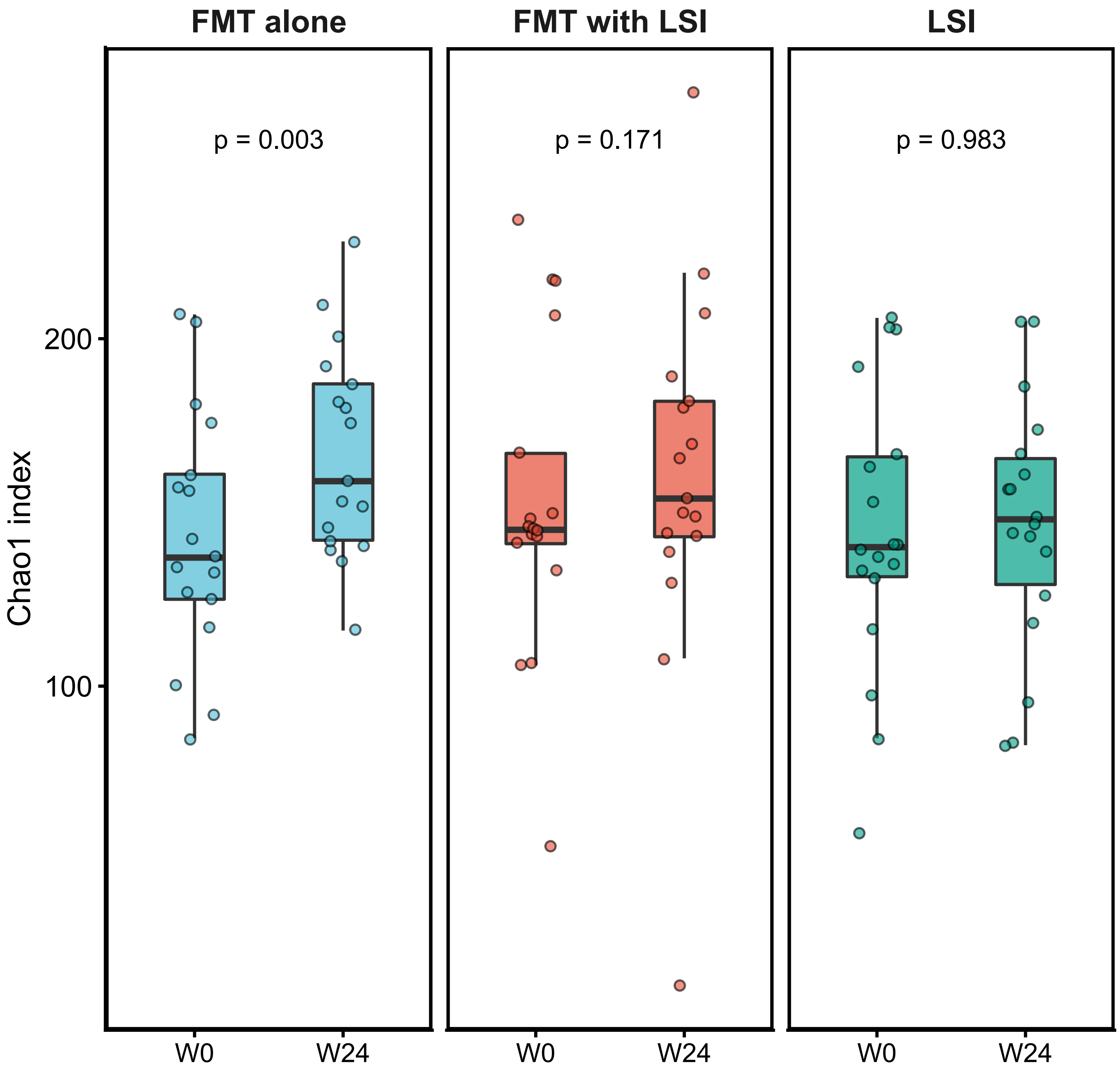

Supplementary Figure 6. Chao1 richness changes at week 24 compared with baseline. Chao1 richness at baseline and week 24 is shown for the FMT alone, FMT with LSI, and sham with LSI groups. Within-group comparisons between baseline and week 24 were performed using paired Wilcoxon tests.

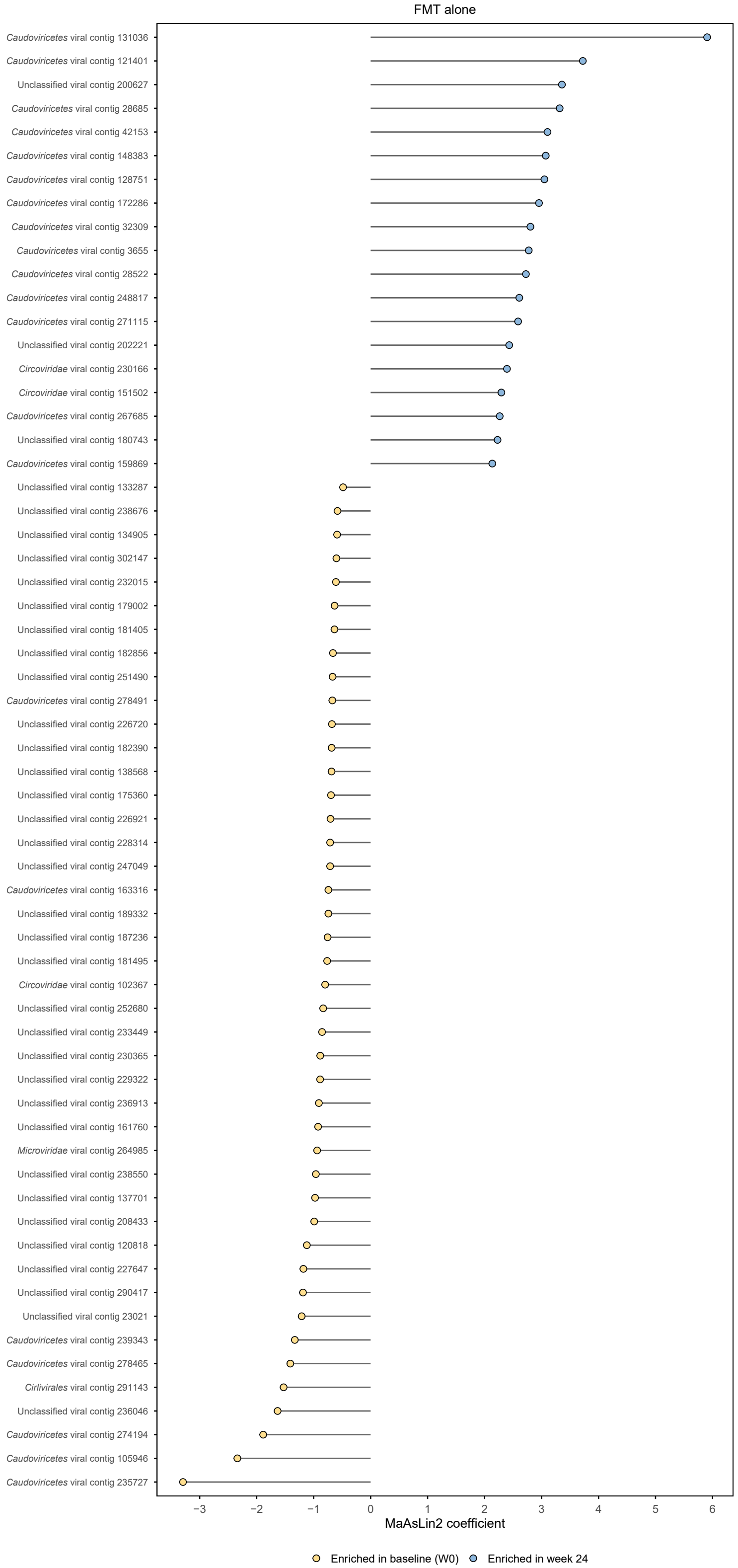

Supplementary Figure 7. Viral contigs significantly changed in the FMT alone group at week 24 vs. baseline. Identified by MaAsLin2, $p<0.05$.

Supplementary Figure 8. Viral contigs significantly changed in the FMT with LSI group at week 24 vs. baseline. Identified by MaAsLin2, p<0.05.

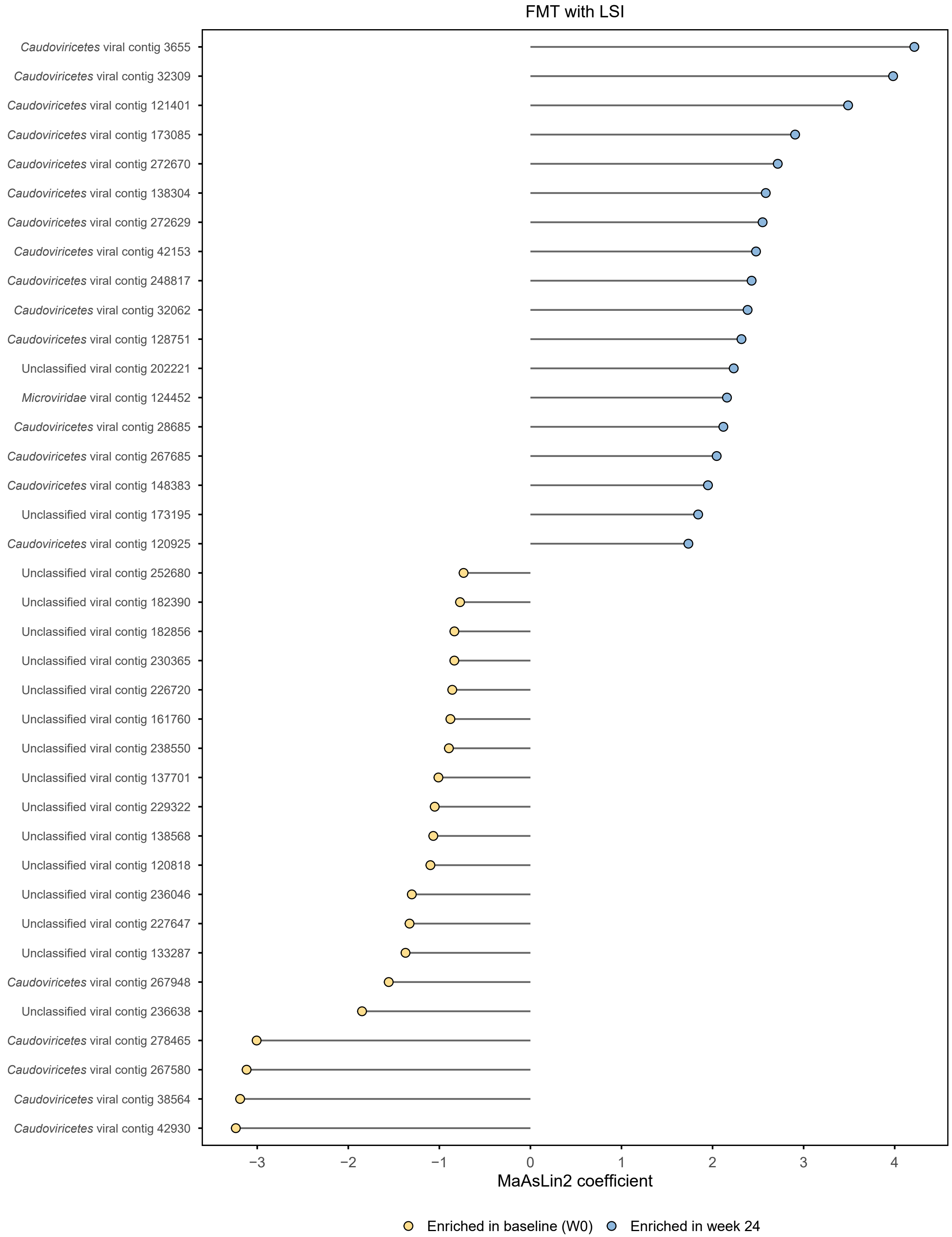

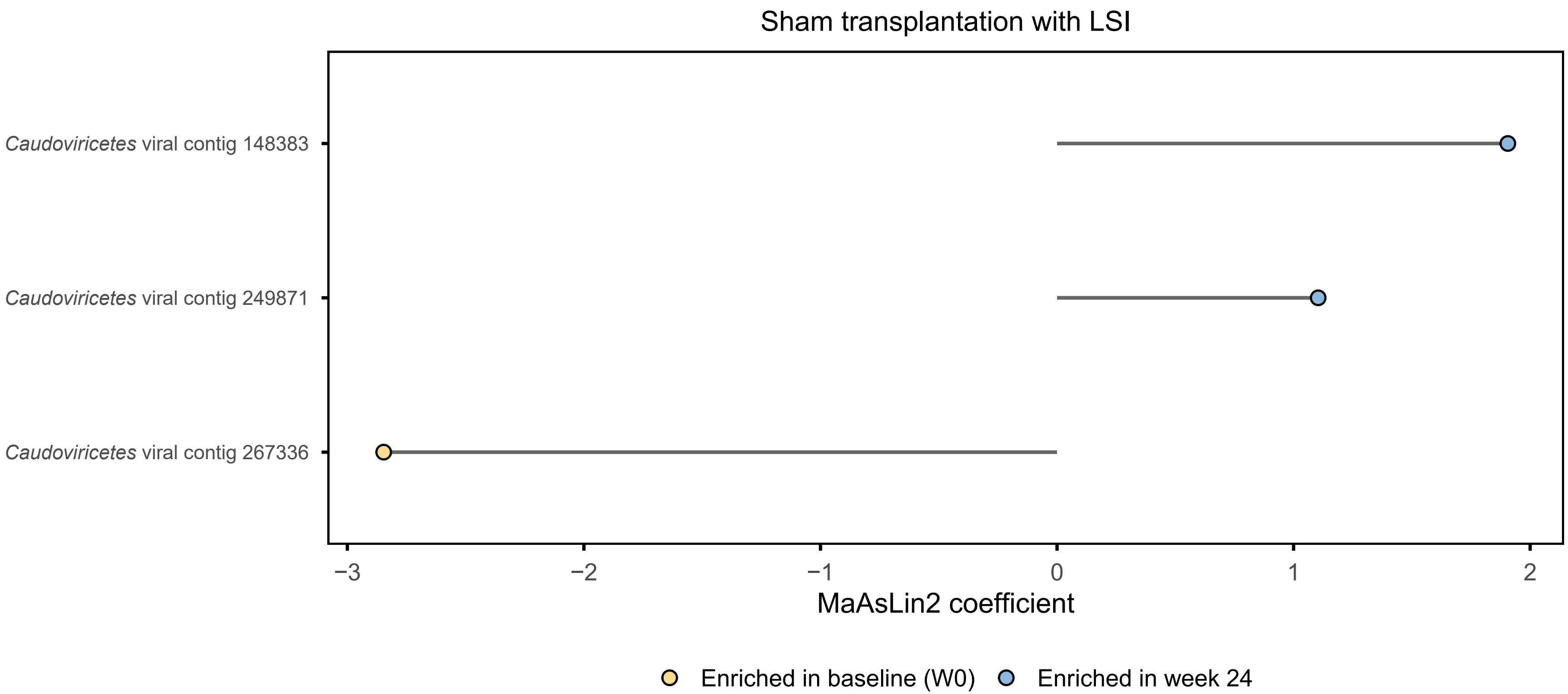

Supplementary Figure 9. Viral contigs significantly changed in the sham with LSI group at week 24 vs. baseline. Identified by MaAsLin2, $p<0.05$.

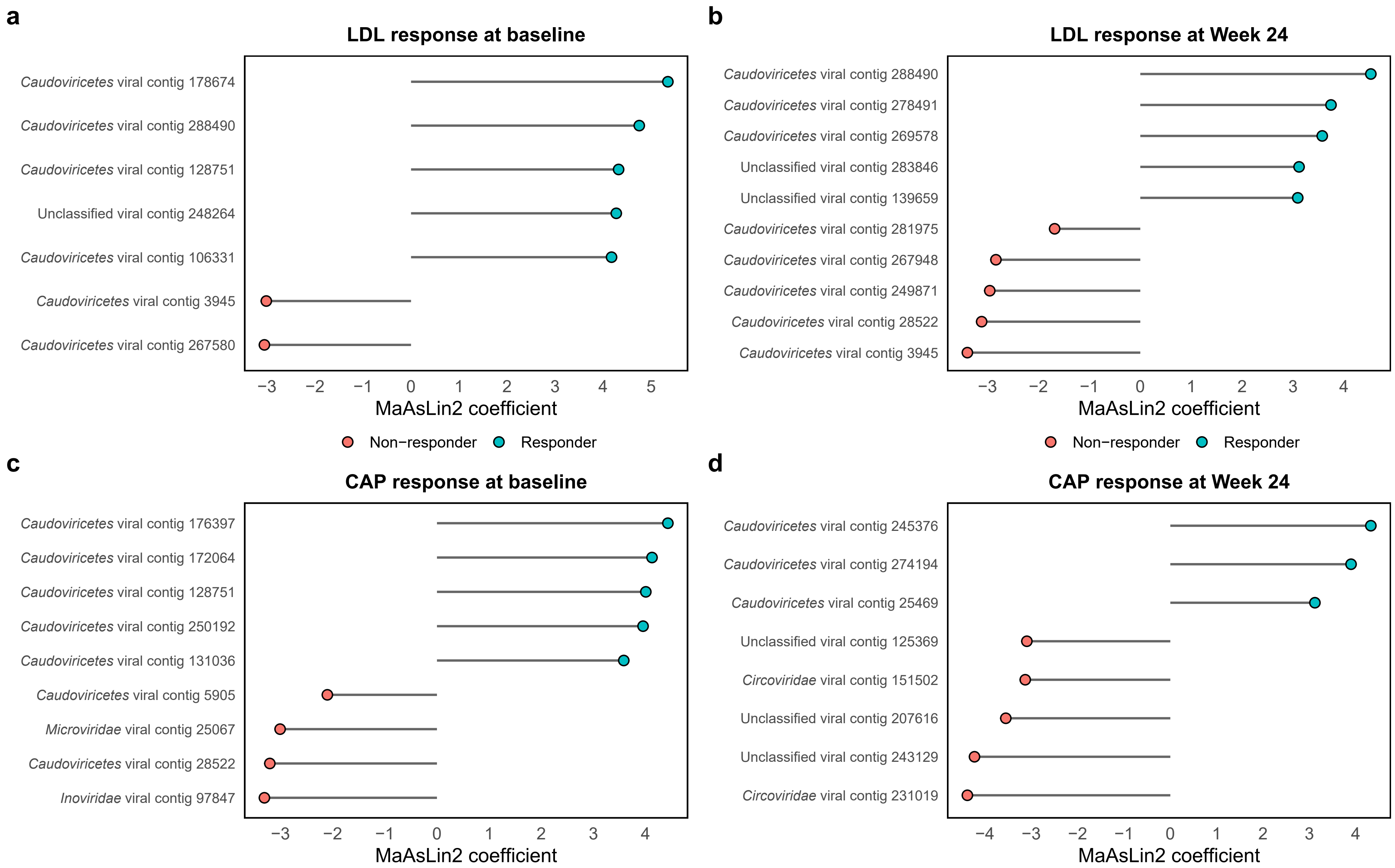

Supplementary Figure 10. Viral contigs associated with differential clinical response to FMT with LSI. Viral contigs associated with LDL response at baseline (a) and week 24 (b), and with liver CAP response at baseline (c) and week 24 (d). Responders (n=8) and non-responders (n=9) were compared using MaAsLin2 ($p<0.05$).
