# Supplementary material for: Gut virome remodeling after fecal microbiota transplantation is associated with virus–bacteria co-variation and metabolic response in obesity and type 2 diabetes
Source: Gut Microbes. 2026 Oct 23;18(1):2746820. doi: 10.1080/19490976.2026.2746820 (PMC13699845; doi:10.1080/19490976.2026.2746820)
Supplement: Supplementary file 2 [file KGMI_A_2746820_SM0694.pdf]

```r
################################################################################
# Supplementary analysis script
# Random Forest workflow for prediction of LDL response
#
# This script reproduces the Random Forest workflow used for
# prediction of LDL response based on virome and bacteriome features.
#
# The workflow includes:
#   1. repeated recursive feature elimination (RFE);
#   2. selection of stable features (selection frequency >=50%);
#   3. five-fold cross-validated Random Forest modelling;
#   4. estimation of Accuracy, Kappa, AUC, Sensitivity,
#      Specificity, and Balanced Accuracy;
#   5. extraction of variable importance.
#
# The same analytical workflow is applied to the virome and
# bacteriome feature matrices.
################################################################################
## 1. Load packages
library(caret)
library(randomForest)
library(pROC)
library(openxlsx)
library(dplyr)
library(tibble)
library(readr)

## 2. Set seeds
RFE_SEED <- 42
FINAL_MODEL_SEED <- 2026

## 3. Import data ---------------------------------------------------------------
# Input matrices should contain samples in rows and features in columns.
# The response factor contain the two classes "Non-responder" and "Responder".
# Feature matrices were generated after prevalence- and abundance-based
# filtering, followed by centered log-ratio (CLR) transformation.
# The virome dataset contains 234 viral contigs, and the bacteriome
# dataset contains 319 bacterial species.
delta_virus <- read.xlsx(
  "delta_virus.xlsx",
  check.names = FALSE
)

delta_bacteria <- read.xlsx(
  "delta_bacteria.xlsx",
  check.names = FALSE
)

# Construct or import y_factor before running the analysis.
# response_metadata <- read_tsv("RF_response_metadata.tsv")
# y_factor <- factor(
#   response_metadata$LDL_response[
#     match(rownames(delta_virus), response_metadata$SampleID)
#   ],
#   levels = c("Non-responder", "Responder")
# )

## 4. Define RFE settings
N_REPEATS <- 50
STABILITY_THRESHOLD <- 50

virus_sizes <- c(
  10, 20, 30, 40, 50, 60, 80, 100, 120, 150, 200, 234
)

bacteria_sizes <- c(
  10, 20, 30, 40, 50, 60, 80, 100, 150, 200, 250, 319
)
```

```r
rf_functions <- rfFuncs

rf_functions$fit <- function(x, y, first, last, ...) {
  randomForest(
    x = x,
    y = y,
    importance = TRUE,
    ntree = 500,
    ...
  )
}

rfe_control <- rfeControl(
  functions = rf_functions,
  method = "cv",
  number = 5,
  verbose = FALSE
)

## 5. Repeated recursive feature elimination
repeat_rfe <- function(
    x,
    y,
    sizes,
    n_repeats = 50,
    seed = 42
) {
  feature_counts <- setNames(
    rep(0, ncol(x)),
    colnames(x)
  )

  accuracy_values <- numeric(n_repeats)
  kappa_values <- numeric(n_repeats)

  set.seed(seed)

  for (i in seq_len(n_repeats)) {
    rfe_result <- rfe(
      x = x,
      y = y,
      sizes = sizes,
      rfeControl = rfe_control
    )

    selected_features <- predictors(rfe_result)

    feature_counts[selected_features] <-
      feature_counts[selected_features] + 1

    best_index <- which.max(rfe_result$results$Accuracy)

    accuracy_values[i] <-
      rfe_result$results$Accuracy[best_index]

    kappa_values[i] <-
      rfe_result$results$Kappa[best_index]
  }

  feature_frequency <- sort(
    feature_counts / n_repeats * 100,
    decreasing = TRUE
  )

  list(
    frequency = feature_frequency,
    mean_accuracy = mean(accuracy_values),
    sd_accuracy = sd(accuracy_values),
    mean_kappa = mean(kappa_values),
```

```
    sd_kappa = sd(kappa_values),
    accuracy_values = accuracy_values,
    kappa_values = kappa_values
  )
}

## 6. Identify stable virome and bacteriome features
virus_rfe <- repeat_rfe(
  x = delta_virus,
  y = y_factor,
  sizes = virus_sizes,
  n_repeats = N_REPEATS,
  seed = RFE_SEED
)

bacteria_rfe <- repeat_rfe(
  x = delta_bacteria,
  y = y_factor,
  sizes = bacteria_sizes,
  n_repeats = N_REPEATS,
  seed = RFE_SEED
)

stable_virus <- names(
  virus_rfe$frequency[
    virus_rfe$frequency >= STABILITY_THRESHOLD
  ]
)

stable_bacteria <- names(
  bacteria_rfe$frequency[
    bacteria_rfe$frequency >= STABILITY_THRESHOLD
  ]
)

if (length(stable_virus) == 0) {
  stop("No stable virome features met the selection-frequency threshold.")
}

if (length(stable_bacteria) == 0) {
  stop("No stable bacteriome features met the selection-frequency threshold.")
}

## 7. Train the final Random Forest model using stable features
train_final_model <- function(
    data,
    outcome,
    selected_features,
    model_name,
    seed = 2026
) {
  selected_data <- data[
    ,
    selected_features,
    drop = FALSE
  ]

  set.seed(seed)

  training_folds <- createFolds(
    outcome,
    k = 5,
    returnTrain = TRUE
  )

  set.seed(seed)

  model_seeds <- vector(
    mode = "list",
```

```r
  length = length(training_folds) + 1
)

for (i in seq_along(training_folds)) {
  model_seeds[[i]] <- sample.int(100000, 3)
}

model_seeds[[length(training_folds) + 1]] <-
  sample.int(100000, 1)

train_control <- trainControl(
  method = "cv",
  number = 5,
  index = training_folds,
  savePredictions = "final",
  classProbs = TRUE,
  seeds = model_seeds,
  allowParallel = FALSE
)

set.seed(seed)

fitted_model <- train(
  x = selected_data,
  y = outcome,
  method = "rf",
  trControl = train_control,
  tuneLength = 3,
  ntree = 500,
  importance = TRUE
)

best_mtry <- fitted_model$bestTune$mtry

best_result <- fitted_model$results[
  fitted_model$results$mtry == best_mtry,
  ,
  drop = FALSE
]

cv_predictions <- fitted_model$pred

if ("mtry" %in% colnames(cv_predictions)) {
  cv_predictions <- cv_predictions %>%
    filter(mtry == best_mtry)
}

roc_result <- roc(
  response = cv_predictions$obs,
  predictor = cv_predictions$Responder,
  levels = c("Nonresponder", "Responder"),
  direction = "<",
  quiet = TRUE
)

confusion_result <- confusionMatrix(
  data = cv_predictions$pred,
  reference = cv_predictions$obs,
  positive = "Responder"
)

importance_table <- varImp(fitted_model)$importance

importance_table$Overall <- apply(
  importance_table,
  1,
  max
)
```

```r
  importance_table <- importance_table %>%
    rownames_to_column("Feature") %>%
    arrange(desc(Overall))

  importance_table$Importance_normalized <-
    importance_table$Overall /
    max(importance_table$Overall) * 100

  list(
    model_name = model_name,
    model = fitted_model,
    feature_count = length(selected_features),
    selected_features = selected_features,
    best_mtry = best_mtry,
    accuracy = unname(best_result$Accuracy),
    kappa = unname(best_result$Kappa),
    auc = as.numeric(auc(roc_result)),
    sensitivity = unname(
      confusion_result$byClass["Sensitivity"]
    ),
    specificity = unname(
      confusion_result$byClass["Specificity"]
    ),
    balanced_accuracy = unname(
      confusion_result$byClass["Balanced Accuracy"]
    ),
    variable_importance = importance_table,
    top10 = head(importance_table, 10),
    cv_predictions = cv_predictions,
    roc = roc_result,
    confusion_matrix = confusion_result
  )
}

## 8. Train virome and bacteriome models
virome_model <- train_final_model(
  data = delta_virus,
  outcome = y_factor,
  selected_features = stable_virus,
  model_name = "Virome",
  seed = FINAL_MODEL_SEED
)

bacteriome_model <- train_final_model(
  data = delta_bacteria,
  outcome = y_factor,
  selected_features = stable_bacteria,
  model_name = "Bacteriome",
  seed = FINAL_MODEL_SEED
)

## 9. Summarize model performance -------------------------------------------
model_comparison <- tibble(
  Model = c("Virome", "Bacteriome"),
  Stable_features = c(
    virome_model$feature_count,
    bacteriome_model$feature_count
  ),
  Accuracy = c(
    virome_model$accuracy,
    bacteriome_model$accuracy
  ),
  Kappa = c(
    virome_model$kappa,
    bacteriome_model$kappa
  ),
  AUC = c(
    virome_model$auc,
    bacteriome_model$auc
```

```
  ),
  Sensitivity = c(
    virome_model$sensitivity,
    bacteriome_model$sensitivity
  ),
  Specificity = c(
    virome_model$specificity,
    bacteriome_model$specificity
  ),
  Balanced_accuracy = c(
    virome_model$balanced_accuracy,
    bacteriome_model$balanced_accuracy
  )
)

model_comparison
################################################################################


################################################################################
# Supplementary analysis script
# Donor-derived virome engraftment analysis
#
# This script reproduces the workflow used to quantify
# donor-derived lean-associated virome engraftment.
#
# Workflow:
#   1. define pooled donor virome;
#   2. identify paired baseline and week-24 recipients;
#   3. calculate donor-derived viral contigs;
#   4. quantify donor-derived virome engraftment;
#   5. compare engraftment rates among treatment groups.
################################################################################
library(dplyr)
library(tidyr)
library(stringr)
library(tibble)

## 1. Define donor samples
donor_samples <- c(
  "D4_S280",
  "D8_S528",
  "D9_BhdST013",
  "D15_BhdST464",
  "D16_BhdST466",
  "D18_BhdST774"
)

## 2. Convert abundance matrix into presence/absence format
virus_long <- virus_relab %>%
  as.data.frame() %>%
  rownames_to_column("virus_id") %>%
  pivot_longer(
    cols = -virus_id,
    names_to = "sample_id",
    values_to = "abundance"
  ) %>%
  filter(abundance > 0) %>%
  select(sample_id, virus_id)

## 3. Construct the pooled donor virome
donor_virus_set <- virus_long %>%
  filter(sample_id %in% donor_samples) %>%
  pull(virus_id) %>%
  unique()

## 4. Identify subjects with paired baseline and week-24 samples
sample_info <- tibble(sampleID = colnames(virus_relab)) %>%
  filter(str_detect(sampleID, "^FDM")) %>%
```

```
  mutate(
    subject = sub("W[0-9]+$", "", sampleID),
    time = sub(".*(W[0-9]+)$", "\\1", sampleID)
  )

has_W0_W24 <- sample_info %>%
  filter(time %in% c("W0", "W24")) %>%
  distinct(subject, time) %>%
  count(subject, name = "n_time") %>%
  filter(n_time == 2) %>%
  pull(subject)

## 5. Prepare metadata
meta_clean <- Clinical_metadata %>%
  mutate(subject_id = studyID) %>%
  filter(
    subject_id %in% has_W0_W24,
    time %in% c("W0", "W24")
  ) %>%
  mutate(
    timepoint = ifelse(time == "W0", "baseline", "post"),
    group = case_when(
      group == "FMT" ~ "FMT alone",
      group == "FMTandLMP" ~ "FMT with LSI",
      group == "LMP" ~ "LSI",
      TRUE ~ NA_character_
    )
  ) %>%
  filter(!is.na(group)) %>%
  select(subject_id, sample_id = sampleID, timepoint, group)

complete_subjects <- meta_clean %>%
  distinct(subject_id, group)

## 6. Calculate donor-derived virome engraftment
# Donor-derived viral contigs were defined as viral contigs
# detected in the pooled donor virome and in the recipient
# at week 24, but absent from the recipient at baseline.
# donor-derived contigs = (Donor ∩ Week24) - Baseline
calc_lean_prop <- function(subject_id){

  baseline_sample <- meta_clean %>%
    filter(
      subject_id == !!subject_id,
      timepoint == "baseline"
    ) %>%
    pull(sample_id)

  post_sample <- meta_clean %>%
    filter(
      subject_id == !!subject_id,
      timepoint == "post"
    ) %>%
    pull(sample_id)

  baseline_virome <- virus_long %>%
    filter(sample_id == baseline_sample) %>%
    pull(virus_id) %>%
    unique()

  post_virome <- virus_long %>%
    filter(sample_id == post_sample) %>%
    pull(virus_id) %>%
    unique()

  donor_derived <- intersect(
    donor_virus_set,
    post_virome
  ) %>%
```

```
    setdiff(baseline_virome)

  length(donor_derived) /
    length(post_virome)
}

## 7. Calculate engraftment for each subject
engraftment_results <- complete_subjects %>%
  rowwise() %>%
  mutate(
    lean_prop = calc_lean_prop(subject_id),
    engraftment_status = ifelse(
      lean_prop >= 0.20,
      "Yes",
      "No"
    )
  ) %>%
  ungroup()

## 8. Summarize engraftment rate
summary_table <- engraftment_results %>%
  group_by(group) %>%
  summarise(
    engraftment_rate =
      mean(engraftment_status == "Yes") * 100,
    responders =
      sum(engraftment_status == "Yes"),
    total = n(),
    .groups = "drop"
  )
################################################################################


################################################################################
# Supplementary analysis script
# MaAsLin2 differential abundance workflow
#
# The workflow includes:
#   1. identification of recipients with paired baseline and week-24 samples;
#   2. selection of donor and recipient baseline samples;
#   3. prevalence- and abundance-based feature filtering;
#   4. centered log-ratio (CLR) transformation;
#   5. differential abundance analysis using MaAsLin2.
#
# The same analytical framework was applied to the other MaAsLin2 analyses
# by modifying the included samples, metadata variables, and contrasts.
################################################################################
## 1. Load packages
library(tidyverse)
library(compositions)
library(Maaslin2)

## 2. Identify recipients with paired baseline and week-24 samples
sample_names <- colnames(virus_relab)

sample_info <- tibble(sample_id = sample_names) %>%
  filter(str_detect(sample_id, "^FDM")) %>%
  mutate(
    subject_id = sub("W[0-9]+$", "", sample_id),
    timepoint = sub(".*(W[0-9]+)$", "\\1", sample_id)
  )

paired_subjects <- sample_info %>%
  filter(timepoint %in% c("W0", "W24")) %>%
  distinct(subject_id, timepoint) %>%
  count(subject_id, name = "n_timepoints") %>%
  filter(n_timepoints == 2) %>%
  pull(subject_id)
```

```
baseline_samples <- sample_info %>%
  filter(
    subject_id %in% paired_subjects,
    timepoint == "W0"
  ) %>%
  pull(sample_id)

## 3. Define donor samples
donor_samples <- c(
  "D4_S280",
  "D8_S528",
  "D9_BhdST013",
  "D15_BhdST464",
  "D16_BhdST466",
  "D18_BhdST774"
)
samples_use <- c(
  donor_samples,
  baseline_samples
)

## 4. Prepare the viral abundance matrix
# The input matrix contains viral contigs in rows and samples in columns.
# The selected matrix is transposed to samples × viral contigs.
virus_matrix <- virus_relab %>%
  select(all_of(samples_use)) %>%
  t() %>%
  as.data.frame()

## 5. Filter viral contigs by abundance and prevalence
# Viral contigs were retained if they had:
#   mean relative abundance >=0.0001; and
#   prevalence >=5% across the included samples.
mean_abundance <- colMeans(
  virus_matrix,
  na.rm = TRUE
)

prevalence <- colMeans(
  virus_matrix > 0,
  na.rm = TRUE
)

retained_contigs <- names(mean_abundance)[
  mean_abundance >= 0.0001 &
    prevalence >= 0.05
]

virus_filtered <- virus_matrix[
  ,
  retained_contigs,
  drop = FALSE
]

## 6. Perform centered log-ratio transformation
# A pseudocount of 1 × 10^-6 was added before CLR transformation.
virus_clr <- clr(
  acomp(virus_filtered + 1e-6)
) %>%
  as.data.frame()

## 7. Prepare metadata
# Metadata should contain one row per sample and a group variable
# distinguishing lean donors from recipient baseline samples.
metadata <- metadata[
  rownames(virus_clr),
  ,
  drop = FALSE
]
```

```r
stopifnot(
  identical(
    rownames(virus_clr),
    rownames(metadata)
  )
)

## 8. Run MaAsLin2
maaslin_fit <- Maaslin2(
  input_data = virus_clr,
  input_metadata = metadata,
  output = "Maaslin2_donor_vs_recipient_baseline",
  fixed_effects = c("group"),
  normalization = "NONE",
  transform = "NONE",
  analysis_method = "LM",
  correction = "BH"
)

## 9. Extract nominally significant viral contigs
maaslin_results <- read_tsv(
  "Maaslin2_donor_vs_recipient_baseline/all_results.tsv",
  show_col_types = FALSE
)

significant_results <- maaslin_results %>%
  filter(
    metadata == "group",
    pval < 0.05
  ) %>%
  select(
    feature,
    value,
    coef,
    pval,
    qval
  ) %>%
  mutate(
    enrichment_direction = ifelse(
      coef < 0,
      "Enriched in lean donors",
      "Enriched in recipient baseline"
    )
  )
################################################################################
```
