# Supplementary material for: Gut virome remodeling after fecal microbiota transplantation is associated with virus–bacteria co-variation and metabolic response in obesity and type 2 diabetes
Source: Gut Microbes. 2026 Oct 23;18(1):2746820. doi: 10.1080/19490976.2026.2746820 (PMC13699845; doi:10.1080/19490976.2026.2746820)
Supplement: Supplementary file 3 [file KGMI_A_2746820_SM0690.docx]

**Supplementary Table S4. Cross-validated performance of the virome and bacteriome random forest models in the primary and sensitivity analyses**

|  | Primary analysis | | Feature-number-matched sensitivity analysis | SMOTE sensitivity analysis | |
| --- | --- | --- | --- | --- | --- |
|  | Virome model | Bacteriome model | Bacteriome model matched | Virome model + SMOTE | Bacteriome model + SMOTE |
| Stable features | N=8 | N=40 | N=8 | N=8 | N=40 |
| Accuracy | 0.848 | 0.814 | 0.786 | 0.814 | 0.819 |
| Kappa | 0.539 | 0.318 | 0.454 | 0.472 | 0.460 |
| AUC | 0.916 | 0.899 | 0.877 | 0.896 | 0.872 |
| Specificity | 0.957 | 1.000 | 0.870 | 0.913 | 0.957 |
| Sensitivity | 0.556 | 0.333 | 0.556 | 0.556 | 0.444 |
| Balanced Accuracy | 0.756 | 0.667 | 0.713 | 0.734 | 0.700 |

Abbreviations: AUC, area under the receiver operating characteristic curve; SMOTE, Synthetic Minority Over-sampling Technique.
SMOTE was applied only within the training portion of each stratified five-fold cross-validation split, while the held-out validation folds retained the original class distribution. The same cross-validation folds were used for the primary and SMOTE sensitivity analyses.
