# Supplementary material for: Gut virome remodeling after fecal microbiota transplantation is associated with virus–bacteria co-variation and metabolic response in obesity and type 2 diabetes
Source: Gut Microbes. 2026 Oct 23;18(1):2746820. doi: 10.1080/19490976.2026.2746820 (PMC13699845; doi:10.1080/19490976.2026.2746820)
Supplement: Supplementary file 4 [file KGMI_A_2746820_SM0692.docx]

**Supplementary Table S2. Baseline characteristics of participants included (n=52) versus excluded (n=9) from the virome analysis**

| Characteristic | Included in virome analysis (n=52) | Excluded from virome analysis (n=9) | *P* value |
| --- | --- | --- | --- |
| Male, n (%) | 38 (73.1) | 5 (55.6) | 0.429 |
| Age, years (P25-P75) | 55.5 (47.0-61.2) | 50.0 (47.0-64.0) | 0.992 |
| Smoker/ex-smoker, n (%) | 14 (26.9) | 4 (44.4) | 0.429 |
| Alcoholic drinker/ex-drinker, n (%) | 5 (9.6) | 2 (22.2) | 0.273 |
| Body weight, kg (P25-P75) | 93.2 (82.1-99.1) | 86.9 (75.0-93.2) | 0.410 |
| BMI, kg/m^2^ (P25-P75) | 33.2 (29.8-35.8) | 30.8 (28.9-35.2) | 0.458 |
| Systolic blood pressure, mmHg (P25-P75) | 136.0 (123.5-143.8) | 140.0 (116.0-159.0) | 0.715 |
| Diastolic blood pressure, mmHg (P25-P75) | 78.0 (72.0-84.0) | 85.0 (75.0-91.0) | 0.247 |
| Waist circumference, cm (P25-P75) | 113.0 (102.6-117.9) | 103.0 (99.5-114.5) | 0.219 |
| Waist-to-hip ratio (P25-P75) | 0.98 (0.94-1.03) | 0.96 (0.93-1.02) | 0.345 |
| Fasting glucose, mmol/L (P25-P75) | 6.3 (5.6-6.7) | 6.1 (5.1-7.0) | 0.784 |
| Fasting insulin, mIU/L (P25-P75) | 18.9 (13.0-28.1) | 19.1 (10.1-32.6) | 0.916 |
| HbA1c, % (P25-P75) | 7.3 (6.6-8.5) | 7.0 (6.5-7.9) | 0.476 |
| Cholesterol |  |  |  |
| Total, mmol/L (P25-P75) | 4.2 (3.6-4.8) | 4.1 (3.8-4.5) | 0.589 |
| LDL, mmol/L (P25-P75) | 2.1 (1.6-2.4) | 1.6 (1.4-2.6) | 0.378 |
| HDL, mmol/L (P25-P75) | 1.3 (1.1-1.5) | 1.5 (1.1-1.5) | 0.361 |
| Triglycerides, mmol/L (P25-P75) | 1.6 (1.2-2.0) | 1.6 (1.0-2.0) | 0.737 |
| ALT, IU/L (P25-P75) | 34.5 (21.8-45.5) | 26.0 (19.0-28.0) | 0.234 |
| ALP, IU/L (P25-P75) | 67.0 (57.0-78.2) | 71.0 (66.0-81.0) | 0.548 |
| Albumin, g/L (P25-P75) | 40.0 (38.0-42.0) | 40.0 (38.0-41.0) | 0.791 |
| Bilirubin, μmol/L (P25-P75) | 12.5 (9.0-14.0) | 11.0 (9.0-14.0) | 0.943 |
| CRP, mg/L (P25-P75) | 1.5 (0.8-4.2) | 1.4 (0.9-2.5) | 0.804 |
| Liver stiffness, kPa (P25-P75) | 7.2 (5.0-8.7) | 5.5 (4.9-7.8) | 0.453 |
| Liver CAP, dB/m (P25-P75) | 342.0 (305.2-366.8) | 321.0 (290.5-358.0) | 0.664 |
| Dietary |  |  |  |
| Energy intake, kcal/day (P25-P75) | 1964.8 (1594.6-2243.5) | 1772.4 (1566.9-1973.0) | 0.602 |
| Protein, g/day (P25-P75) | 89.1 (71.3-105.3) | 63.6 (59.1-79.9) | 0.219 |
| Carbohydrate, g/day (P25-P75) | 209.0 (175.5-239.0) | 204.1 (176.5-210.9) | 0.391 |
| Fiber, g/day (P25-P75) | 11.3 (8.8-14.2) | 9.0 (6.9-11.4) | 0.219 |
| Sugar, g/day (P25-P75) | 40.4 (27.0-57.8) | 19.8 (10.5-39.3) | 0.274 |
| Total fat, g/day (P25-P75) | 82.5 (61.2-96.5) | 74.5 (67.0-89.9) | 0.801 |
| Saturated fat, g/day (P25-P75) | 20.9 (14.1-24.4) | 16.5 (14.2-19.8) | 0.469 |
| Sodium, mg/day (P25-P75) | 4000.8 (3313.5-4615.3) | 4745.4 (3924.7-5423.3) | 0.337 |

Abbreviations: ALP, alkaline phosphatase; ALT, alanine aminotransferase; BMI, body mass index; CAP, controlled attenuation parameter; CRP, C-reactive protein; HbA1c, glycated hemoglobin; HDL, high-density lipoprotein; LDL, low-density lipoprotein.
Data are expressed as number of subjects (%) or median (P25-P75), as appropriate. Continuous variables were compared using the Mann-Whitney U test, and categorical variables were compared using Fisher’s exact test, as appropriate. Two-sided P values <0.05 were considered statistically significant.
