# Supplementary material for: Gut virome remodeling after fecal microbiota transplantation is associated with virus–bacteria co-variation and metabolic response in obesity and type 2 diabetes
Source: Gut Microbes. 2026 Oct 23;18(1):2746820. doi: 10.1080/19490976.2026.2746820 (PMC13699845; doi:10.1080/19490976.2026.2746820)
Supplement: Supplementary file 5 [file KGMI_A_2746820_SM0696.docx]

**Supplementary Table S1. Baseline characteristics of the 52 participants included in the virome analysis**

| Characteristic | FMT with LSI (n=17) | FMT alone (n=17) | LSI (n=18) |
| --- | --- | --- | --- |
| Male, n (%) | 13 (76.5) | 12 (70.6) | 13 (72.2) |
| Age, years (P25-P75) | 56.0 (43.0-62.0) | 54.0 (45.0-59.0) | 56.5 (50.8-62.5) |
| Smoker/ex-smoker, n (%) | 7 (41.2) | 4 (23.5) | 3 (16.7) |
| Alcoholic drinker/ex-drinker, n (%) | 4 (23.5) | 0 (0.0) | 1 (5.6) |
| Body weight, kg (P25-P75) | 96.1 (81.0-103.9) | 91.3 (84.5-99.3) | 92.9 (82.7-94.4) |
| BMI, kg/m^2^ (P25-P75) | 30.2 (29.0-34.0) | 34.2 (30.0-36.6) | 33.4 (32.5-33.9) |
| Systolic blood pressure, mmHg (P25-P75) | 136.0 (129.0-143.0) | 138.0 (117.5-143.5) | 130.5 (125.2-146.0) |
| Diastolic blood pressure, mmHg (P25-P75) | 78.0 (70.5-82.0) | 77.0 (72.0-84.0) | 76.5 (70.2-84.0) |
| Waist circumference, cm (P25-P75) | 104.8 (100.0-115.2) | 113.8 (103.4-118.5) | 111.2 (106.5-117.6) |
| Waist-to-hip ratio (P25-P75) | 0.95 (0.93-1.02) | 1.00 (0.98-1.03) | 0.98 (0.95-1.06) |
| Fasting glucose, mmol/L (P25-P75) | 6.4 (5.6-6.8) | 6.4 (5.8-7.6) | 6.2 (5.5-6.6) |
| Fasting insulin, mIU/L (P25-P75) | 21.2 (14.9-33.5) | 15.8 (12.7-23.0) | 18.9 (14.9-29.0) |
| HbA1c, % (P25-P75) | 7.1 (6.7-8.4) | 7.5 (6.3-8.2) | 7.6 (6.6-8.7) |
| Cholesterol |  |  |  |
| Total, mmol/L (P25-P75) | 4.5 (3.8-5.0) | 4.4 (3.6-4.9) | 4.0 (3.5-4.4) |
| LDL, mmol/L (P25-P75) | 2.3 (2.0-2.9) | 2.2 (1.8-2.3) | 1.7 (1.5-2.3) |
| HDL, mmol/L (P25-P75) | 1.4 (1.2-1.5) | 1.2 (1.1-1.5) | 1.2 (1.0-1.5) |
| Triglycerides, mmol/L (P25-P75) | 1.3 (1.1-1.8) | 1.8 (1.2-2.7) | 1.5 (1.2-2.4) |
| ALT, IU/L (P25-P75) | 34.0 (23.0-41.0) | 36.0 (20.0-47.0) | 32.0 (21.2-46.5) |
| ALP, IU/L (P25-P75) | 58.0 (51.0-68.0) | 71.0 (63.0-79.0) | 69.0 (58.5-77.5) |
| Albumin, g/L (P25-P75) | 40.0 (38.0-43.0) | 39.0 (38.0-42.0) | 40.0 (37.0-42.0) |
| Bilirubin, μmol/L (P25-P75) | 11.0 (9.0-13.0) | 10.0 (9.0-13.0) | 13.0 (10.5-14.0) |
| CRP, mg/L (P25-P75) | 1.7 (0.7-3.9) | 2.4 (0.8-3.2) | 1.2 (0.8-5.8) |
| Liver stiffness, kPa (P25-P75) | 5.6 (4.7-7.8) | 7.2 (5.8-8.2) | 7.7 (6.0-9.5) |
| Liver CAP, dB/m (P25-P75) | 345.0 (323.0-360.0) | 351.0 (277.0-369.0) | 334.0 (310.2-374.2) |
| Medication use |  |  |  |
| Lipid-lowering medication use, n (%) | 11 (64.7) | 12 (70.6) | 12 (66.7) |
| Antidiabetic medication use, n (%) | 9 (52.9) | 9 (52.9) | 6 (33.3) |
| Dietary |  |  |  |
| Energy intake, kcal/day (P25-P75) | 1967.1 (1722.3-2212.5) | 1982.8 (1646.3-2328.9) | 1904.5 (1594.6-2243.5) |
| Protein, g/day (P25-P75) | 76.0 (65.9-106.1) | 89.8 (83.7-115.5) | 86.4 (74.4-95.4) |
| Carbohydrate, g/day (P25-P75) | 216.1 (182.2-241.1) | 202.1 (173.8-229.7) | 221.5 (172.6-247.0) |
| Fiber, g/day (P25-P75) | 11.1 (8.6-13.6) | 10.4 (8.7-13.6) | 12.7 (10.5-15.0) |
| Sugar, g/day (P25-P75) | 45.3 (36.4-59.4) | 40.0 (25.1-59.6) | 35.7 (27.3-51.5) |
| Total fat, g/day (P25-P75) | 84.0 (68.2-101.5) | 80.9 (67.5-99.8) | 86.3 (57.7-92.4) |
| Saturated fat, g/day (P25-P75) | 22.5 (14.4-23.8) | 20.6 (18.1-25.1) | 20.5 (12.9-24.7) |
| Sodium, mg/day (P25-P75) | 3800.7 (3292.3-4729.2) | 4130.4 (3309.7-4371.0) | 3999.5 (3449.7-4421.2) |

Abbreviations: ALP, alkaline phosphatase; ALT, alanine aminotransferase; BMI, body mass index; CAP, controlled attenuation parameter; CRP, C-reactive protein; FMT, fecal microbiota transplantation; HbA1c, glycated hemoglobin; HDL, high-density lipoprotein; LDL, low-density lipoprotein; LSI, lifestyle intervention.

Data are expressed as number of subjects (%) or median (P25-P75), as appropriate. Baseline characteristics are presented descriptively by randomized treatment group; no between-group significance testing was performed. Dietary variables were derived from baseline 3-day dietary records.
