# Supplementary material for: Gut virome remodeling after fecal microbiota transplantation is associated with virus–bacteria co-variation and metabolic response in obesity and type 2 diabetes
Source: Gut Microbes. 2026 Oct 23;18(1):2746820. doi: 10.1080/19490976.2026.2746820 (PMC13699845; doi:10.1080/19490976.2026.2746820)
Supplement: Supplementary file 6 [file KGMI_A_2746820_SM0698.docx]

**Supplementary Table S3. Lipid-lowering and antidiabetic medication use and changes according to LDL response status among FMT recipients included in the LDL-response analysis**

| Variable | Non-responder (n=23) | Responder (n=9) | Fisher P |
| --- | --- | --- | --- |
| Lipid-lowering medication use | 19/23 | 4/9 | **0.075** |
| Lipid-lowering change | 1/19 | 0/4 | **1.000** |
|  |  |  |  |
| Antidiabetic medication use | 12/23 | 5/9 | **1.000** |
| Antidiabetic change | 5/12 | 2/5 | **1.000** |

Abbreviations: FMT, fecal microbiota transplantation; LDL, low-density lipoprotein.

Data are expressed as number of participants with medication use or change / total number of participants in the corresponding group. Comparisons between LDL responders and non-responders were performed using Fisher’s exact test. Two-sided P values <0.05 were considered statistically significant.
